# Resistance to the nucleotide analogue cidofovir in HPV(+) cells: a multifactorial process involving UMP/CMP kinase 1

**DOI:** 10.18632/oncotarget.7006

**Published:** 2016-01-25

**Authors:** Dimitri Topalis, Tatiane C. Nogueira, Tim De Schutter, Chahrazade El Amri, Marcela Krečmerová, Lieve Naesens, Jan Balzarini, Graciela Andrei, Robert Snoeck

**Affiliations:** ^1^ Rega Institute for Medical Research, KU Leuven, 3000 Leuven, Belgium; ^2^ Sorbonne Universités, UPMC University Paris 06, UMR 8256, B2A, Biological Adaptation and Ageing, Integrated Cellular Ageing and Inflammation, Molecular and Functional Enzymology, Paris, 75252 Paris Cedex 05, France; ^3^ Institute of Organic Chemistry and Biochemistry, Academy of Sciences of The Czech Republic, v.v.i., CZ-166 10 Prague 6, Czech Republic

**Keywords:** human papillomavirus, cervical carcinoma, UMP-CMP kinase, cidofovir, NTP metabolism

## Abstract

Human papillomavirus (HPV) is responsible for cervical cancer, and its role in head and neck carcinoma has been reported. No drug is approved for the treatment of HPV-related diseases but cidofovir (CDV) exhibits selective antiproliferative activity.

In this study, we analyzed the effects of CDV-resistance (CDV^R^) in two HPV(+) (SiHa_CDV_ and HeLa_CDV_) and one HPV(−) (HaCaT_CDV_) tumor cell lines. Quantification of CDV metabolites and analysis of the sensitivity profile to chemotherapeutics was performed. Transporters expression related to multidrug-resistance (MRP2, P-gp, BCRP) was also investigated.

Alterations of CDV metabolism in SiHa_CDV_ and HeLa_CDV,_ but not in HaCaT_CDV,_ emerged *via* impairment of UMP/CMPK1 activity. Mutations (P64T and R134M) as well as down-regulation of UMP/CMPK1 expression were observed in SiHa_CDV_ and HeLa_CDV,_ respectively. Altered transporters expression in SiHa_CDV_ and/or HeLa_CDV,_ but not in HaCaTCDV, was also noted.

Taken together, these results indicate that CDV^R^ in HPV(+) tumor cells is a multifactorial process.

## INTRODUCTION

Cidofovir (CDV) is an acyclic nucleoside phosphonate (ANP) with broad spectrum anti-DNA virus activity [1]. Its intravenous form is approved for therapy of cytomegalovirus retinitis in AIDS patients [2]. CDV is also used off-label for treatment of several diseases associated with herpesviruses other than cytomegalovirus, comprising adeno-, pox-, polyoma-, and papillomaviruses [3–6]. Besides its well-characterized antiviral activity, CDV is active as an antitumor agent in several animal models, including human cervical carcinoma xenografts in athymic nude mice [7–10]. CDV inhibitory effect on tumor growth has been attributed to its recognition by cellular DNA polymerases and incorporation into genomic DNA causing DNA breaks and/or apoptosis induction [11]. The higher sensitivity of cervical carcinoma cells carrying the human papillomavirus (HPV) genome, i.e. SiHa [HPV16 (+)] and HeLa [HPV18 (+)] to CDV compared to primary human keratinocytes (PHKs) has been explained by the differential response of tumor cells and normal cells to DNA damage [12, 13].

The cytosolic UMP/CMP kinase 1, a key enzyme in the activation of antiviral and anticancer drugs, catalyzes the phosphorylation of (d)CMP analogues to their diphosphate forms [14–16]. 2′-deoxycytidine (dC) (i.e. lamivudine and emtricitabine) or 2′-deoxycytidine monophosphate (dCMP) (i.e. CDV) analogues require phosphorylation by UMP/CMPK1 [17]. The first and last phosphorylation steps of cytidine 2′-deoxynucleoside analogues are catalyzed, respectively, by deoxycytidine kinase (dCK) and nucleoside diphosphate kinase (NDPK) [17]. Similarly, the anticancer agents cytarabine (araC) and gemcitabine (dFdC) are activated by UMP/CMPK1 following prior conversion to their 5′-monophosphate forms by dCK [18]. The mitochondrial form of UMP/CMPK (i.e. UMP/CMPK2) possesses distinct substrate specificity since it recognizes dUMP and dCMP as best phosphate acceptors while the cytosolic form preferentially phosphorylates CMP and UMP [19]. Both enzymes are also able to phosphorylate nucleotide analogues such as the monophosphate forms of the anti-cancer agents araC-MP and dFdC-MP [15, 18–20].

Failure to anticancer therapy is often due to drug-resistance because of emergence of mutations in the cellular target or in one of the activating enzymes, multidrug-resistance (MDR) events leading to efflux of the active metabolite, or activation of DNA repair pathways [21–23]. Several drug-resistance mechanisms for araC or dFdC were described, including i) deamination by cytidine deaminase which converts araC into the inactive metabolite araU [24, 25], ii) down-regulation of the equilibrative nucleoside transporter (hENT1), the major carrier for araC and dFdC uptake [26], iii) circumvention of dCK-dependent araC and dFdC phosphorylation by down-regulation of dCK expression or point mutations leading to weaker activation of araC and dFdC [27, 28].

Unlike araC and dFdC, CDV intracellular uptake occurs via fluid-phase endocytosis and is independent of hENT [29]. In primary kidney tubular cells, CDV uptake is mediated through the organic anion transporters OAT1, and to a lesser extent OAT3. CDV activation is independent from dCK since CDV bears a phosphonate moiety at its methoxyhydroxypropyl part, bypassing then the initial phosphorylation by a nucleoside kinase. Thus, CDV-resistance (CDV^R^) mechanisms are expected to be different from those described for cytosine nucleoside analogues.

In this study, we investigated the impact of CDV^R^ on UMP/CMPK1 and on nucleoside metabolism, in particular on CTP and UTP biosynthesis. Three cell lines [two HPV(+) (SiHa and HeLa) and one HPV(−) (HaCaT)] were selected *in vitro* for resistance to CDV and were denoted SiHa_CDV,_ HeLa_CDV_ and HaCaT_CDV._ The susceptibility of these cell lines to several chemotherapeutics was assessed, as well as, the emergence of multi-drug-resistance mechanisms through upregulation of specific transporters. The metabolism of CDV, and in particular its incorporation into genomic DNA, was also investigated in these CDV^R^ cell lines.

## RESULTS

### Growth rate of CDV^R^ cells and sensitivity to ANPs

SiHa_CDV_ and HeLa_CDV_ had a significantly slower growth rate than parental cells [doubling time (DT) of 36 h *versus* 22 h and 24 h *versus* 21 h, respectively] (Figure S1). In contrast, cellular growth rates were not significantly different between HaCaT_parental_ and HaCaT_CDV_ cells (i.e. DT of 26 h and 23 h, respectively).

**Figure 1:**
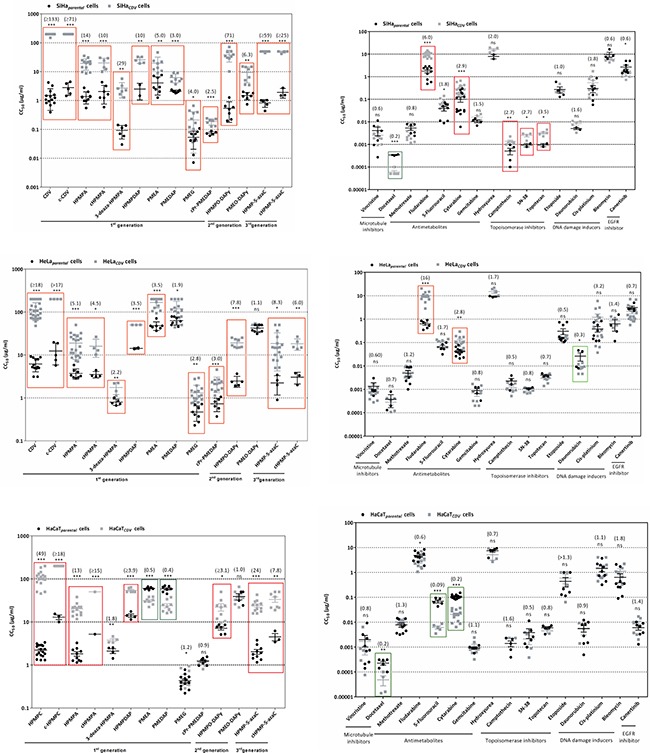
Sensitivity of parental and CDV^R^ SiHa, HeLa and HaCaT cells to different ANPs and other chemotherapeutics Between parentheses are presented the fold resistance values for each compound.

To determine the sensitivity of parental and CDV^R^ cells to CDV, CC_50_'s following 7 days of incubation in the presence of the drug were evaluated (Figure 1). The highest fold-resistance (FR) was found for SiHa_CDV_ (> 133) while for HeLa_CDV_ and HaCaT_CDV_, FR values were of > 18 and > 49, respectively.

The sensitivity of CDV^R^ cells to several chemotherapeutics was also investigated (Figure 1). Five different levels of resistance/hypersensitivity were recognized when considering a statistical significant difference (*p* < 0.05) between compounds' CC_50_ values for parental and CDV^R^ cells together with FR values: high resistance (FR ≥ 10), moderate resistance (5 ≤ FR < 10), mild resistance (2.0 ≤ FR < 5), no resistance (0.4 < FR < 2.0) and hypersensitivity (0.4 ≥ FR).

Regarding ANPs, SiHa_CDV_ cells were found to be highly resistant to HPMP derivatives with a FR in the range of 10 to 133 and moderate resistant to PME derivatives (Figure 1). For HeLa_CDV_ cells, high resistance was observed for cHPMPC, moderate resistance to HPMPO-DAPY, HPMP-5azaC, cHPMP-azaC and HPMPA, while mild resistance was found for 3-deaza-HPMPA, PMEA, PMEG and cPr-PMEDAP. HaCaT_CDV_ was highly resistant to cHPMPC, HPMPA, cHPMPA, HPMP-5azaC, moderately resistant to cHPMP-5azaC and exhibited mild resistance to HPMPDAP and HPMPO-DAPY. Hypersensitivity to PMEDAP and PMEA was observed for HaCaT_CDV_.

### Sensitivity of CDV^R^ cells to distinct chemotherapeutic agents

The results for other chemotherapeutics, structurally unrelated to ANPs but possessing antiproliferative activity against several type of cancers, are shown in Figure 1. Moderate resistance was observed against fludarabine and mild resistance against cytarabine, camptothecin, SN-38 and topotecan, while hypersensitivity was demonstrated to docetaxel when tested on the SiHa_CDV_. HeLa_CDV_ was found to have high resistance to fludarabine and mild resistance to cytarabine, while hypersensitivity was found for daunorubicin. HaCaT_CDV_ was shown to be hypersensitive to docetaxel, 5-fluorouracil and cytarabine.

Microarray data highlighted some genes likely involved in hypersensitivity or resistance to different chemotherapeutics in CDV^R^ cells. For some of the differentially expressed genes, in particular those coding for proteins involved in uptake/efflux of different chemotherapeutics, enzymes required for their activation or catabolism and their target proteins, protein level variations were indicated (Table 1).

**Table 1:** Genes that might be involved in resistance or hypersensitivity to antiproliferative drugs in SiHa (A), HeLa (B) and HaCaT (C)

A	Docetaxel	Fludarabine	Cytarabine	Camptothecin	SN-38	Topotecan
**SiHa_CDV_**	**Hypersensitivity** *MAP2* (−1.30) *MAPT* (−1.00)	**Resistance** *SLC29A1*(−1.59)	**Resistance** *CMPK1*(mutations) (↓protein) *NT5E* (+ 2.20)	**Resistance** *ABCC2* (+ 2.17) (↑protein) *ABCG2* (+ 1.02) (↑protein)	**Resistance** *ABCC2* (+ 2.17) (↑protein) *ABCG2* (+ 1.02) (↑protein)	**Resistance** *ABCG2* (+ 1.02) (↑protein) *TOP2A* (+ 1.02)

B	Fludarabine	Cytarabine	Daunorubicin
**HeLa_CDV_**	**Resistance** *AK2* (−1.19) *POLE4* (+ 1.15)	**Resistance** *CMPK1* (−1.29) (↓protein) *NT5E* (+ 1.08)	**Hypersensitivity** ? genes involved in DNA damage response

C	Docetaxel	5-Fluorouracil	Cytarabine
**HaCaT_CDV_**	**Hypersensitivity** *TUBB2A* (−1.01) *KIF1C* (−1.24) *KIF3C* (−1.27) *ABCC3* (−2.86) *CYP1B1* (−1.05) *CYP3A7* (−2.77)	**Hypersensitivity** *SLC29A2* (+ 1.47) *ABCC3* (−2.86)	**Hypersensitivity** *CMPK1* (−0.31) (≉protein) *NT5E* (−1.00)

Abbreviations: *TUBB2A*: tubulin beta 2A, *MAP2*: microtubule associated protein 2, *MAPT*: microtubule associated protein tau, *KIF1C*: kinesin like protein 1C, *KIF3C*: kinesin like protein 3C, *CYP1B1*: cytochrome P450 1B1, *CYP3A7*: cytochrome P450 3A7, *SLC29A1/A2*: equilibrative nucleoside transporter 1/2, *AK2*: adenylate kinase 2, *POLE4*: DNA polymerase epsilon subunit 4, *ABCG2*: ATP binding cassette type G2, *NT5E*: ecto-5′-nucleotidase (CD73), *CMPK1*: UMP/CMP kinase 1, *TOP2A*: topoisomerase II A, *ABCC2/3*: ATP binding cassette type C2/C3.

Note: Between parentheses are shown the fold change (log_N_ FC) of the upregulated (+) and downregulated (−) genes. For resistance or hypersensitivity consideration, the cut-off was set to 2.5 (for resistance) and 0.4 (for hypersensitivity). ↑, ↓ and ≉ symbolize, respectively, increase, decrease and unchanged level of protein expression as observed by Western blot.

In SiHa_CDV_, cross-resistance to camptothecin, SN-38 and topotecan can be explained *via* up-regulation of efflux pump genes *ABCG2* (BCRP) and/or *ABCC2* (MRP2) (Table 1), as demonstrated in previous studies with different malignant cells [30–32]. Down-regulation of influx transporters *SLC22A6* (OAT1) and *SLC29A1* (ENT1), and decreased expression of phosphorylating enzymes (*AK2* and *CMPK1*), may be responsible for cross-resistance to fludarabine and cytarabine, respectively.

In HeLa_CDV_, up-regulation of the DNA polymerase ε sub-unit 4 (*POLE4*) (a subunit responsible for activation of replication and repair) might explain cross-resistance to fludarabine (Table 1) because of increased excision of fludarabine incorporated into DNA [33]. Hypersensitivity to docetaxel, 5-FU and cytarabine in HaCaT_CDV_ could be mediated through up-regulation of influx transporters (*SLC29A2*; ENT2) or proteins involved in drug catabolism such as *CYP1B1* and *CYP3A7*.

### Differential protein expression of MRP2, BCRP, P-gp and OAT1 transporters

**Figure 2:**
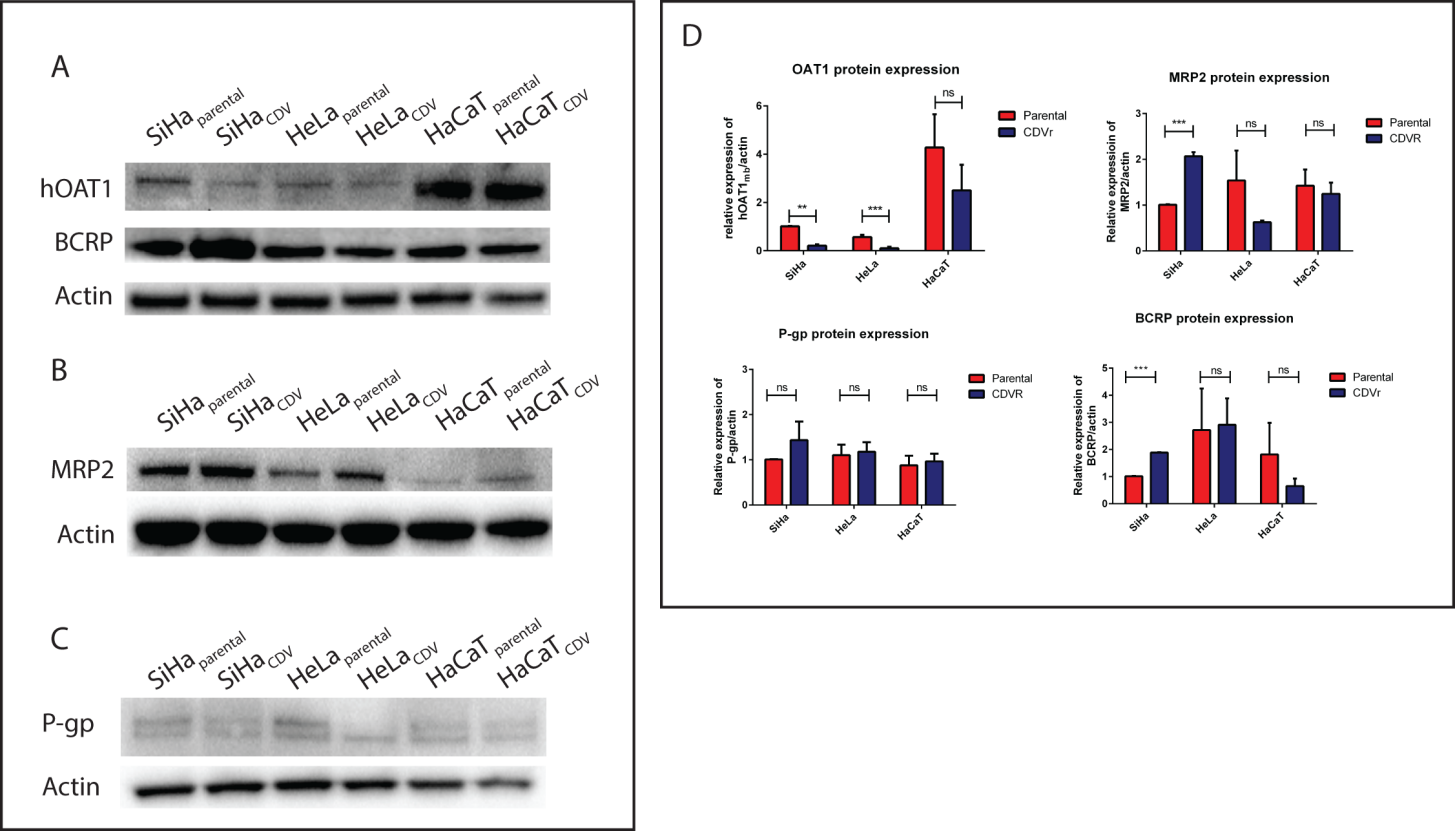
Differential expression of several ABC and SLC transporters Western blot detection was performed to measure the levels of OAT1 and BCRP (**A**), MRP2 (**B**), and P-gp (**C**). Quantification of each band was done and standardized using actin as housekeeping gene (**D**).

The differential expression of several transporters involved in multidrug-resistance [i.e. MRP2 (*ABCC2*), P-gp/MDR1 (*ABCB1*), BCRP (*ABCG2*) and OAT1 (*SLC22A6*)] was measured by Western blot. OAT1 was downregulated in SiHa_CDV_ and HeLa_CDV_ (2-fold) but not in HaCaT_CDV_ (Figure 2A–2D). BCRP levels were increased in SiHa_CDV_ but not in HeLa_CDV_ and HaCaT_CDV_ (Figure 2A–2D). MRP2 was only upregulated in SiHa_CDV_ (Figure 2B–2D) while P-gp was not affected in any of the three CDV^R^ cell lines (Figure 2C–2D).

The sensitivity of parental and CDV^R^ cells to different MDR or P-gp inhibitors was then assessed (Figure S2). Zosuquidar and Tariquidar (P-gp inhibitors) as well as Reversan and MK-571 (MRP inhibitors) act as competitive inhibitors while verapamil and indomethacin are non-competitive inhibitors of MRP1. SiHa_CDV_ showed hypersensitivity to reversan but not to MK-571. HeLa_CDV_ displayed hypersensitivity to reversan and verapamil while HaCaT_CDV_ showed hypersensitivity to tariquidar and indomethacin.

We next evaluated CDV and HPMPA antiproliferative activities in presence of MDR or P-gp inhibitors to rule out differences in transport of purine and pyrimidine analogues (Figure S3). SiHa_parental_ was less sensitive to CDV and HPMPA in presence of the MRP inhibitor MK-571. In HeLa_CDV_, the HPMPA CC_50_ value was lower in presence of 4 μg/ml of zosuquidar than without inhibitor. The other inhibitors did not revert the resistant phenotype. These data point to some alterations of MDR and/or P-gp transporters in SiHa_CDV_ and HeLa_CDV_ cells but not in HaCaT_CDV_.

### Intracellular CDV metabolism

**Figure 3:**
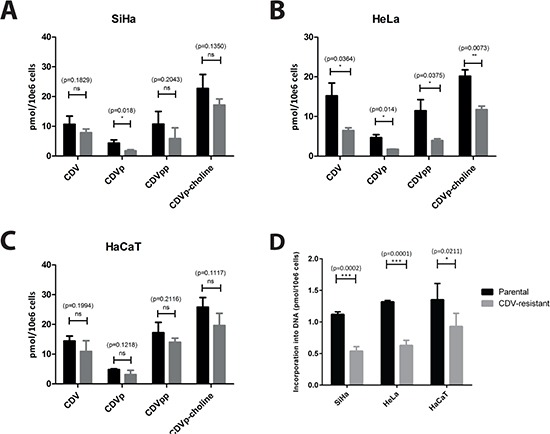
Intracellular metabolism of CDV in parental and CDV^R^ SiHa (A), HeLa (B) and HaCaT (C) cells The intracellular levels of CDV, CDV monophosphate (CDVp), CDV diphosphate (CDVpp) and CDVp-choline have been measured using radiolabeled CDV. The mean values were calculated from at least three independent experiments. (**D**) The figure shows the incorporation of CDV into DNA for the three different cell types. The mean values have been calculated from two independent experiments.

The impact of drug-resistance acquisition on CDV metabolism was analyzed to detect alterations in CDV activation by cellular kinases. In SiHa_CDV_ cells, reduced amounts of CDV, CDV monophosphate (CDVp), CDV diphosphate (CDVpp) (the active drug form) and CDVp-choline were measured compared to SiHa_parental_ cells, but only the decrease in CDVp was statistically significant (Figure 3A). Interestingly, the incorporation of CDV into cellular DNA, measured after a drug-exposure of 24 h, was reduced by 50% in SiHa_CDV_ cells (*p* = 0.0002) (Figure 3D). A more pronounced decrease in CDV activation was observed in HeLa_CDV_ cells (*p* < 0.05 for all four metabolites) (Figure 3B) and CDV incorporation into DNA was also reduced by 50% (*p* = 0.0006). In contrast, in HaCaT_CDV_ cells, no significant differences with parental cells were measured for CDV metabolites while, for CDV incorporation into DNA, a decrease of 30% was observed (Figure 3C and 3D). Hence, each CDV^R^ cell type exhibited a different pattern of CDV activation compared to parental counterpart.

### Levels of UMP/CMPK1 affect CTP and UTP synthesis

**Figure 4:**
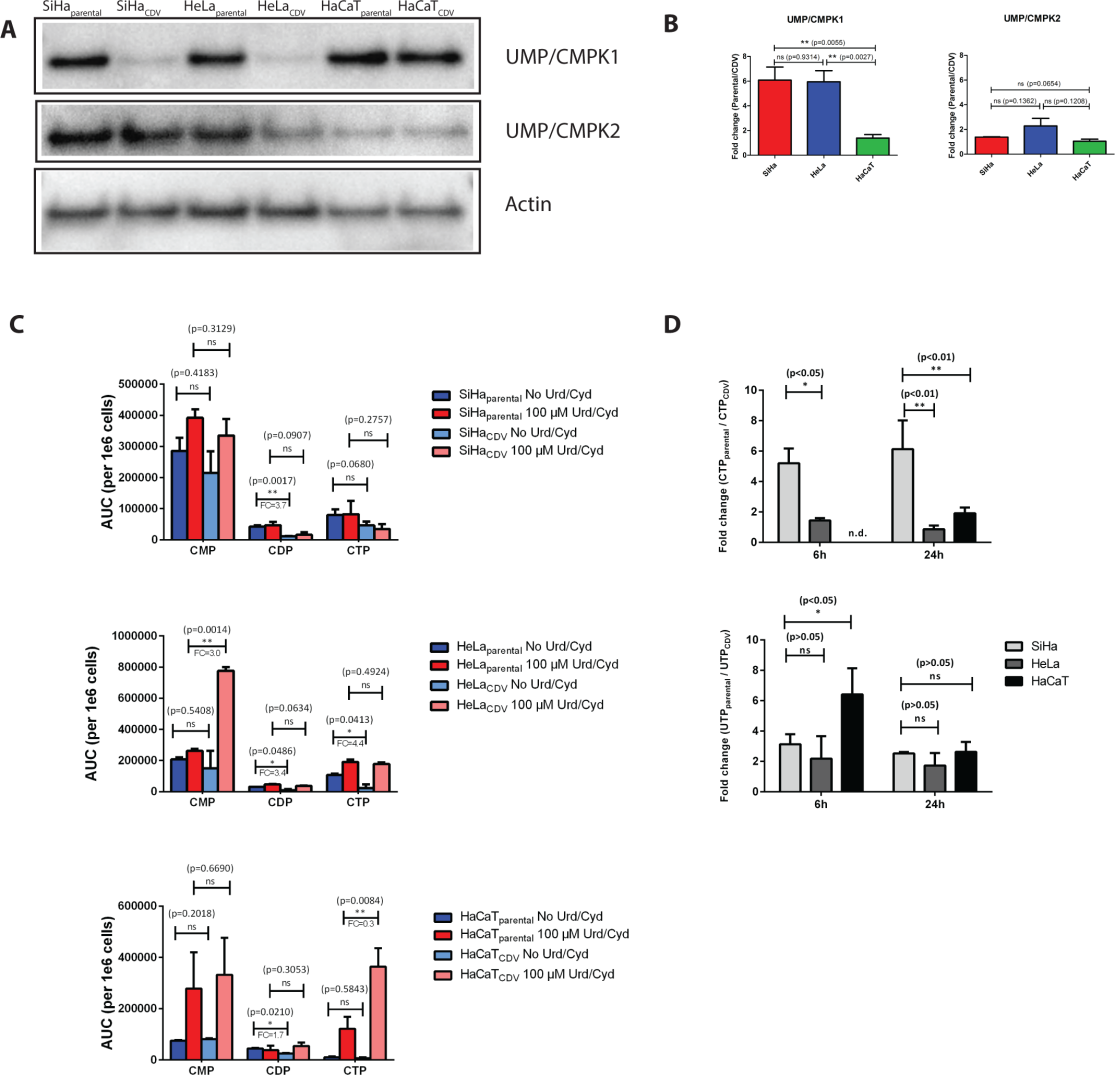
(**A**) Western blot detection of UMP/CMPK1 and 2 in the parental and CDV^R^ SiHa, HeLa and HaCaT cells. (**B**) Fold-change (parental/CDV) of UMP/CMPK1 and 2 gene expression based on relative quantification compare to actin gene expression. The bars indicate the statistical difference between SiHa, HeLa and HaCaT regarding UMP/CMPK1 downregulation. (**C**) Determination of CMP, CDP and CTP levels in parental and CDV^R^ cells by HPLC. Fold change is indicated when the difference in level of nucleotides between the parental and the CDV^R^ cell is statistically significant. (**D**) Ratio of parental over CDV^R^ cells CTP and UTP levels determined by means of HPLC following radioactive cytidine and uridine supplementation. Radiolabeled nucleosides where added to the cell medium and CTP and UTP levels were measured after 6 h and 24 h. No CTP level was detected in HaCaT_CDV_ cells, preventing the comparison to the parental cells.

After analyzing protein expression of several influx/efflux pumps in CDV^R^ lines, we next measured the expression of UMP/CMPK1, the enzyme responsible for the first phosphorylation of CDV, in order to detect any possible impairment in the activation pathway of the drug. Parental SiHa and HeLa cells expressed 6-fold more UMP/CMPK1 protein levels than the corresponding CDV^R^ cells (*p* < 0.05) (Figure 4A). In contrast, the difference in UMP/CMPK1 expression between HaCaT_parental_ and HaCaT_CDV_ was only of 1.4-fold (*p* = 0.3679) (Figure 4B). For mitochondrial UMP/CMPK2, the fold-change between parental and CDV^R^ cells was not statistically significant for any of the three cell lines.

The effects of changes on UMP/CMPK1 expression on CTP and UTP synthesis were investigated. CDP levels were significantly higher in parental cells than in CDV^R^ SiHa and HeLa cells (3.4 to 3.7 fold-change) while in HaCaT cells, this difference was only of 1.7 (Figure 4C). These results are in line with the lower levels of UMP/CMPK1 in SiHa_CDV_ and HeLa_CDV_ cells but not in HaCaT_CDV_ cells (Figure 4A–4B). In parallel, the levels of ATP and GTP were also measured in parental and CDV^R^ cell lines (Figure S4). ATP levels were not significantly affected, in the tested conditions, in the different cell lines (Figure S4A) while, regarding GTP levels, a significant increased level was only measured in HaCaT_CDV_ cells compared to the parental counterparts (Figure S4B).

Since UMP/CMPK1 activity is impaired in SiHa and Hela CDV^R^ cells, uridine (Urd) and cytidine (Cyd) were added to the growth medium to restore CTP and UTP levels (Figure 4C). By supplementing the cells with 100 μM of Urd and Cyd, the difference in CDP levels between parental and CDV^R^ cells was abolished. Interestingly, the 4-fold lower CTP levels in HeLa_CDV_ cells than in parental cells was reverted by the addition of Urd/Cyd. In HeLa_CDV_ cells, accumulation of CMP in the presence of Urd/Cyd was found while there was barely any difference in CMP level for HeLa_parental_ cells with or without Urd/Cyd-supplements. Higher CTP levels in Urd/Cyd supplemented HaCaT_CDV_ cells were observed than in the parental cells.

When radiolabeled Cyd and Urd were added to the cells and the levels of newly synthesized CTP and UTP were measured, CTP production was found to be higher in SiHa_parental_ than in SiHa_CDV_ (5-fold and 6-fold difference at 6 h and 24 h post-treatment, respectively). For HaCaT and HeLa cells, the fold-changes in CTP production at 24 h were 1.9 and 0.9, respectively (Figure 4D). These data confirm a more pronounced difference in CTP production between SiHa_CDV_ and SiHa_parental_ cells than in the two other cell types. The UTP level variation seemed similar in the three cell types when measured at 24 h post-treatment (Figure 4D).

### Molecular basis for resistance to CDV: identification of mutations in UMP/CMPK1

Two amino acid changes in the *UMP/CMPK1* of SiHa_CDV_ cells were identified (Figure S5): P64T [in the mobile nucleotide monophosphate (NMP) binding domain] and R134M [in the LID domain] (Figure S6). A mixed population of wild-type or double-mutated UMP/CMPK1, or two single mutated UMP/CMPK1 were found. Unlike SiHa_CDV_ cells, no mutations in UMP/CMPK1 were detected in HeLa_CDV_ and HaCaT_CDV_ cells. No mutations in the *UMP/CMPK2* gene were observed in the CDV^R^ cells. We also genotyped the two HPV oncogenes, i.e. E6 and E7, in SiHa_CDV_ and HeLa_CDV_ cells, but no differences were observed between parental and CDV^R^.

### Enzymatic activity of recombinant UMP/CMPK1

To investigate the impact of the mutations identified in the UMP/CMPK1 of SiHa_CDV_ cells, site-directed mutagenesis was performed on a plasmid containing the coding sequence for UMP/CMPK1 to produce mutant enzymes carrying the P64T, R134M or a combination of both amino acid changes. The enzyme bearing the P64T substitution had impaired enzymatic activity compared to the wild-type protein. Using CMP and UMP as phosphate acceptor, the turn-over number *k*_cat_ was, respectively, 0.31 s^−1^ and 0.35 s^−1^ for the P64T mutant compared to, respectively, 125 s^−1^ and 188 s^−1^ for the wild-type enzyme (Table 2). The affinity (*K*_M_) for these substrates was also affected (*K*_M_ values of 123 μM and 250 μM for, respectively, CMP and UMP with the P64T UMP/CMPK1 mutant, instead of 20 μM and 100 μM with the wild-type enzyme). As a result, the catalytic efficiency (*k*_cat_/*K*_M_) for phosphorylation decreased in the range of 1,340- to 2,480-fold for the mutant P64T UMP/CMPK1. The phosphorylation of dCMP and dUMP was also dramatically affected by the P64T substitution. dCMP was poorly phosphorylated by the P64T mutant, and dUMP was not metabolized at all under the experimental conditions used. The mutant P64T UMP/CMPK1 did not metabolize CDV and its 5-aza derivative (5azaCDV). The phosphorylation of these drugs by the wild-type enzyme was significantly slower than the rate seen for dCMP (*k*_cat_ of 0.09 s^−1^ and 0.47 s^−1^, for CDV and 5azaCDV, respectively *versus* 13.4 s^−1^). On the contrary, cytarabine monophosphate (araCMP) was phosphorylated by the mutant enzyme but with a dramatic decrease of the catalytic rate (*k*_cat_ = 0.019 s^−1^ and *K*_M_ = 327 μM, instead of *k*_cat_ = 130 s^−1^ and *K*_M_ = 315 for wild-type enzyme).

**Table 2:** Kinetic parameters (k_cat_, K_M_ and k_cat_/K_M_) of the wild-type and the recombinant UMP/CMP kinases

	k_cat_ (s^−1^)				K_M_ (μM)				k_cat_/K_M_ (M^−1^s^−1^)			
	WT	P64T	R134M	P64T R134M	WT	P64T	R134M	P64T R134M	WT	P64T	R134M	P64T R134M
**CMP**	**125 ± 20**	**0.31 ± 0.04**	*n.d.*	*n.d.*	**20 ± 5**	**123 ± 50**	*n.d.*	*n.d.*	**6.25 × 106**	**2.52 × 103**	*n.d.*	*n.d.*
**UMP**	**188 ± 10**	**0.35 ± 0.10**	*n.d.*	*n.d.*	**100 ± 25**	**250 ± 25**	*n.d.*	*n.d.*	**1.88 × 106**	**1.4 × 103**	*n.d.*	*n.d.*
**dCMP**	**13.4 ± 4.2**	**0.036 ± 0.010**	*n.d.*	*n.d.*	**110 ± 50**	**970 ± 220**	*n.d.*	*n.d.*	**1.22 × 105**	**37**	*n.d.*	*n.d.*
**dUMP**	**5.80 ± 1.6**	*n.d.*	*n.d.*	*n.d.*	**1034 ± 250**	*n.d.*	*n.d.*	*n.d.*	**5.61 × 103**	*n.d.*	*n.d.*	*n.d.*
**CDV**	**0.09 ± 0.04**	*n.d.*	*n.d.*	*n.d.*	**2300 ± 100**	*n.d.*	*n.d.*	*n.d.*	**40**	*n.d.*	*n.d.*	*n.d.*
**5azaCDV**	**0.47 ± 0.04**	*n.d.*	*n.d.*	*n.d.*	**2080 ± 250**	*n.d.*	*n.d.*	*n.d.*	**226**	*n.d.*	*n.d.*	*n.d.*
**araCMP**	**130 ± 5**	**0.019 ± 0.007**	*n.d.*	*n.d.*	**315 ± 45**	**327 ± 30**	*n.d.*	*n.d.*	**4.1 × 105**	**58**	*n.d.*	*n.d.*

Abbreviations: (n.d. not detected under our experimental conditions).

The enzymes bearing the R134M amino acid change alone or in combination with the P64T, did not exhibit enzymatic activity with the natural substrates nor with CDV. The catalytic efficiencies obtained for the UMP/CMPK1, could be ranked from the best acceptor substrate to the weakest, as follows: CMP > UMP > araCMP > dCMP > dUMP > 5azaCDV > CDV for the wild-type enzyme, and CMP > UMP > araCMP ≥ dCMP for the mutant P64T enzyme. For the wild-type enzyme, the kinetic parameters obtained for the natural substrates were similar to those obtained in a previous study [15].

### Impact of point mutations on UMP/CMPK1 conformation and stability

The effect of the two mutations on the stability of the UMP/CMPK1 protein was assessed by circular dichroism (CD). Far-UV CD spectra of wild-type UMP/CMPK1 without ligand showed the characteristic shape of a α-helix-rich protein with minima at 208 nm and 222 nm and a maximum at 192 nm. The CD spectrum of mutant P64T UMP/CMPK1 showed significant changes in the minima 208 nm and 222 nm but the overall shape is indicating a strong α-helix content (Figure S7A). The effect of the amino acid change P64T is directly reflected on this α-helix environment since the minima at 208 nm and 222 nm appeared to be changed in comparison to the wild-type enzyme. The ratio [θ]_222_/[θ]_208_ evolved from 1.006 to 0.809, indicating a change in the folding of the UMP/CMPK1 protein induced by the P64T amino acid change (θ, being the degree of ellipticity).

The stability of the wild-type and the three mutant UMP/CMPK1 proteins was also investigated by thermal denaturation at two different temperatures (i.e. 5°C and 70°C). Figure S7B shows the Δε at these temperatures for the wild-type and the three UMP/CMPK1 mutants. The P64T mutant started at a less native state compared to the wild-type protein, but at 70°C, the enzyme was not totally unfolded. The R134M mutant was very sensitive to thermal denaturation and at high temperature, the denaturation state was comparable to that of the wild-type enzyme. The double mutant P64T + R134M was at an intermediate state between P64T and R134M (Figure S7B).

The R134M and P64T + R134M UMP/CMPK1 mutants are more sensitive to heat denaturation, suggesting that the P64T substitution triggers important conformational changes, higher stability of the enzyme and markedly impaired activity regarding the natural substrates CMP, UMP, dCMP and dUMP. The mutation R134M totally inactivated the enzyme and, in addition, destabilized the secondary structure, leading to a temperature-sensitive protein.

### Modeling studies of the P64T and R134M amino acid changes

**Figure 5:**
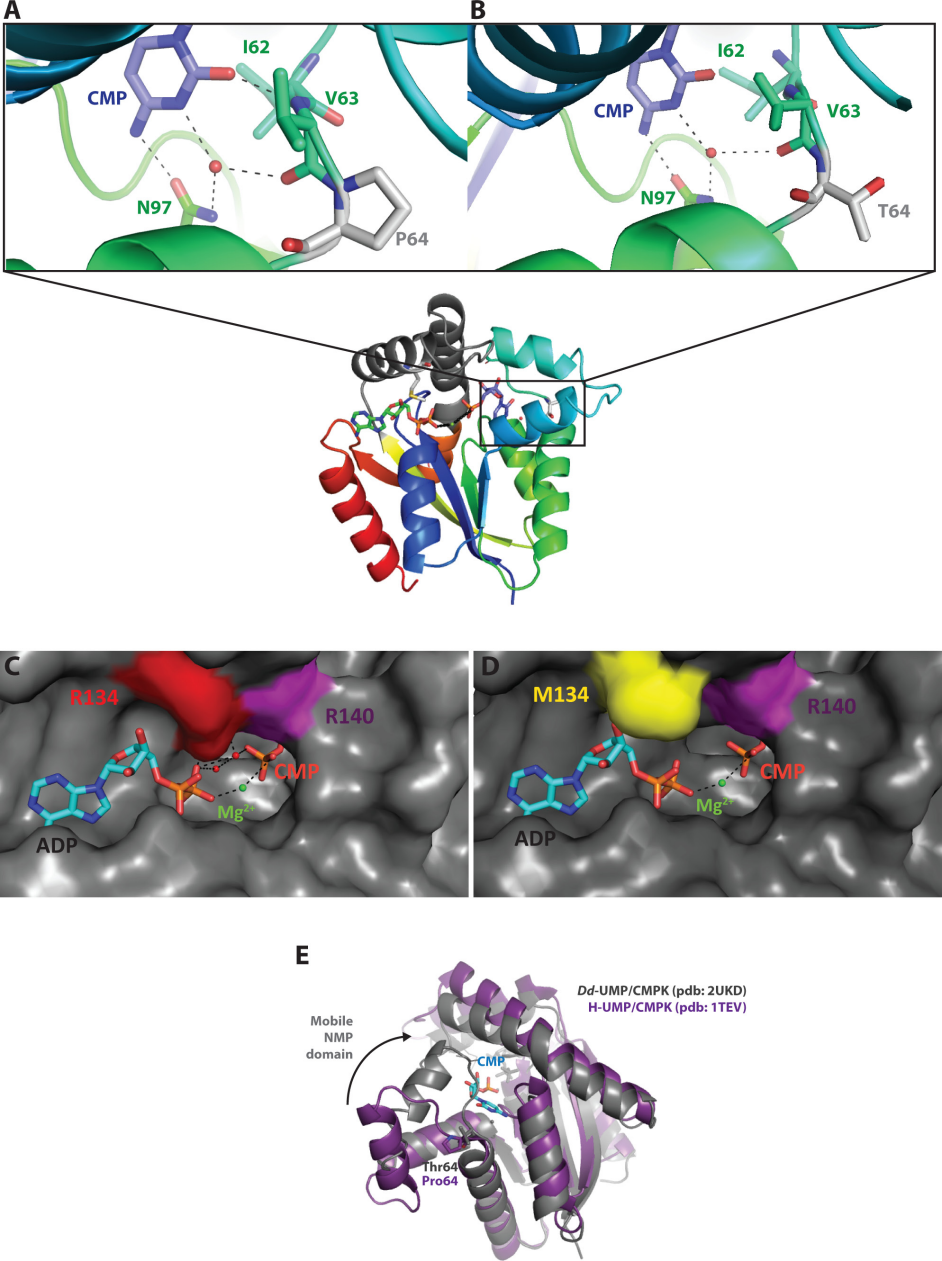
Structure-function relationship analysis of the mutant UMP/CMPK1s (**A**–**B**) Comparison of the NMP binding site of WT and P64T UMP/CMPK1s complexed to CMP (in blue). Mutation at position 64 is represented as a grey residue. Amino acids in interaction with the base of CMP are depicted in green; the black dashes represent the polar interactions between the active site and CMP. (**C**–**D**) ATP binding site of WT and R134M UMP/CMPK1s with position 134 colored in red. ADP, bound to the active site, is colored in blue. Magnesium ion is represented as green sphere. The surface representation of the active site shows the steric hindrance of amino acid change R134M on ADP binding. (**E**) Superposition of the UMP/CMPK1 in open (in purple) and closed (in grey) conformation, showing the important movement that the NMP binding domain undergoes upon CMP binding.

Models of the human UMP/CMPK1 were built using as template the three-dimensional structure of the *Dictyostelium discoideum* UMP/CMPK in the closed conformation complexed with ADP and CMP (pdb code: 2UKD). The cytosine base interacts via water-mediated hydrogen bonds and hydrophobic interactions with residues Ile-62, Val-63 and Asn-97 (Figure 5A). Pro-64 is located next to Ile-62 and Val-63 and allows the positioning of these residues close to the cytosine moiety for the establishment of efficient interactions. The P64T substitution may modify the distance between Ile-62, Val-63 and CMP, and therefore reduce the intensity or abolish the interactions observed in the wild-type UMP/CMPK1 at this position (Figure 5B). The Arg-134 is located in the LID domain, a mobile domain that contains positively charged residues able to stabilize the phosphate of the ATP with the magnesium ion in the active site (Figure 5C). The amino acid change Arg-to-Met at position 134 might dramatically decrease the number of polar interactions between the ATP and CMP molecules and the magnesium ion (Figure 5D). The typical interactions established between an arginine to bridge the phosphate of ATP and CMP for the enzymatic reaction are abolished in the presence of Met-134. As observed with the enzymatic studies, this mutation results in inactivation of the enzyme. The superposition between the open conformation of the human UMP/CMPK1 and the closed conformation of the *Dictyostelium discoideum* UMP/CMPK bound to CMP clearly shows that the helix α4 undergoes an important movement while closing the active site (Figure 5E). The residue at position 64 is located at the hinge, triggering this important conformational change and, consequently, an amino acid change at this specific position might have an important effect on the catalytic activity of the enzyme. This hypothesis is consistent with the kinetic parameters obtained with the mutant P64T UMP/CMPK1 that exhibited slower rates of phosphorylation for the natural substrates CMP, UMP and dCMP.

## DISCUSSION

Emergence of drug-resistance after long-term exposure to antiproliferative agents is frequently observed in anticancer therapy. In general, drug-resistance can be related to several mechanisms including decreased uptake of the drug, slower drug-activation and faster drug-excretion via efflux pump(s). Studies of cytarabine and gemcitabine resistance mechanism have played an important role in the characterization of pyrimidine metabolism in tumor cells, but up to now, no description of resistance has been done regarding UMP/CMPK1. This is the first report of mutations in the UMP/CMPK1 linked to drug-resistance.

In SiHa_CDV_ cells, two mutations were identified in the C*MPK1* gene leading to low levels of UMP/CMPK1 protein. On the other hand, the reduced UMP/CMPK1 protein level in HeLa_CDV_ was linked to down-regulation of C*MPK1* gene expression. In HaCaT_CDV_, no changes in UMP/CMPK1 protein content were detected. These results are in line with impairment of CDV metabolism in SiHa_CDV_ and HeLa_CDV_ but not in HaCaT_CDV_. These data highlight a different pattern of CDV metabolism in each CDV^R^ cell lines and that reduction of UMP/CMPK1 protein level is not enough to explain CDV^R^, since HaCaT_CDV_ cells exhibited CDV^R^ despite no impairment of UMP/CMPK1.

Reduced levels of UMP/CMPK1 in SiHa_CDV_ resulted in poor CDV activation as shown by a significant decrease in CDVp but not in CDVpp or CDVp-choline. The enzymatic UMP/CMPK1 assay demonstrated that both amino acid changes (i.e. P64T and R134M) abolished the capacity of the enzyme to efficiently phosphorylate CDV. However, we did detect intracellular CDVp and the subsequent metabolites CDVpp and CDVp-choline in SiHa_CDV_, suggesting that both wild-type and double mutant UMP/CMPK1s are expressed in these cells. The electropherograms of UMP/CMPK1 cDNA sequencing performed for SiHa_CDV_ cells at passages 55 and 230 showed a mixed population of wild-type and mutant cells (1:1 ratio) at both positions (Figure S5). Thus, it can be inferred that both mutations are present in one allele and the other allele encodes for a wild-type enzyme. Co-existence of a mixed population of cells, one expressing exclusively wild-type enzyme and the other one expressing only mutant UMP/CMPK1, appears to be highly improbable since SiHa_parental_ cells have a faster growth rate and, hence, would overgrow SiHa_CDV_ cells after several passages. Furthermore, the enzymatic study performed with mutant proteins showed that both P64T and R134M single mutations lead to impaired or non-functional enzymes while SiHa_CDV_ cells were able to generate CTP from exogenous Cyd, UTP from added Urd, as well as CDVp and subsequent metabolites from CDV. Quantification of UMP/CMPK1 in SiHa_CDV_ cells showed a dramatic decrease in the protein pool in comparison with parental cells. Protein stability assays showed that the P64T + R134M UMP/CMPK1 mutant is highly sensitive to temperature denaturation, and hence is an instable protein. Since the mutant enzyme is less stable, its intracellular half-life might be altered leading to faster degradation via the proteasome pathway. In conclusion, these data indicate that SiHa_CDV_ cells most likely express both wild-type and mutant UMP/CMPK1.

The impact of altered UMP/CMPK1 expression on the activation of (d)C analogues might differ from cell to cell since it has been reported that modulation of UMP/CMPK1 expression did not influence the activation of C and U analogues (dFdC, L-OddC and 5-FU) in RKO cells but well that of 2FdC, araC and 5-FU in HeLa S3 and HCT8 cells [34,35]. In our study, we observed that genes related to metabolism of nucleoside analogues, such as *CMPK1* and *SLC29A1*, were differentially expressed in SiHa_CDV_ compared to SiHa_parental_ cells. However, these alterations were not linked to a drug-resistance phenotype such as gemcitabine-resistance. We cannot exclude that impairment of gemcitabine uptake and activation could be compensated by other transporters and enzymes (e.g. SLC 29A2 and CMPK2). Furthermore, no data for the cell lines here studied have been published yet describing the uptake and activation of cytarabine, gemcitabine and CDV.

In HeLa_CDV_ cells, the total CDV metabolism seemed to be affected since not only the amount of CDVp was reduced but also CDV, CDVpp and CDVp-choline pools. The impairment in the whole metabolism of CDV might imply a role for the uptake or efflux considering the reduced amounts of intracellular CDV and CDVpp, resulting in a decreased CDV incorporation into the genomic DNA.

In HaCaT_CDV_, no significant differences were observed regarding CDV metabolism and a small decrease in CDV incorporation into DNA was noted although a 58-fold resistance to CDV was found. Thus, other events may be responsible for CDV^R^ in these cells, such as alterations in CDV-targeted cellular pathways. The microarray analysis performed on parental and CDV^R^ HaCaT cells showed that DNA repair mechanism(s) might also be responsible for CDV^R^. The activation of these pathways might explain the resistance to CDV even if no significant impairment of the metabolism of this drug was registered. Furthermore, other cellular pathways (such as ‘Wnt/β-catenin signaling’, ‘ERK/MAPK signaling’, ‘EGF signaling’, ‘NFkB signaling’ or ‘acute phase response signaling’) might have an effect on CDV^R^ since several genes are differentially expressed in the HaCaT_CDV_ compared to HaCaT_parental_.

The kinetic parameters obtained with the recombinant P64T UMP/CMPK1 enzyme with natural substrates indicated that the apparent affinity *K*_M_ was not dramatically affected. The main reason for the impaired phosphorylation of natural substrates for this mutant enzyme was the rate of catalysis since the *k*_cat_ values were 403-, 537- and 372-fold slower for CMP, UMP and dCMP, respectively, than for the wild-type enzyme. The mobility of the NMP domain, induced by substrate binding to the active site, appears to play an important role in the catalysis reaction [36]. Substrate binding leads to displacement of 18 Å of E59 carbon α, a residue belonging to the NMP binding domain [37]. The P64 residue, located in the hinge, allows the NMP binding site main helices (α3–α4) to change conformation in order to stabilize the closed conformation of the enzyme. The amino acid change P64T may alter this mobility, affecting the rate of phosphorylation by UMP/CMPK1 of any phosphate acceptor.

Circular dichroism assays performed in thermal denaturing conditions demonstrated that the mutant P64T protein has a higher stability than the wild-type enzyme. By decreasing the mobility of the NMP domain, the P64T substitution may lead to a more compact protein. Even though the mutant enzyme acquires a higher stability, its enzymatic activity is dramatically impaired and CDV phosphorylation totally abolished.

The second affected position, R134, is located in the LID domain and is crucial for the activity of the UMP/CMPK1 enzyme since it interacts with ATP. The arginine to methionine change at the homologous position R132 in the human AMP kinase (that is structurally related to UMP/CMPK1) reduces the phosphorylation rate of AMP from 650 s^−1^ to 0.08 s^−1^ [38]. This conserved residue has an important role in the enzymatic activity of nucleoside monophosphate kinases, since it stabilizes the transition state for phosphate transfer between the two substrates. Circular dichroism analysis of the mutants bearing the R134M amino acid change revealed that the mutant protein has higher instability than the wild-type one, consistent with reduced protein levels.

Several multidrug-resistance proteins have been shown to be upregulated in CDV^R^ cells. Regarding ANPs, MRP4, -5, -7 and -8 were shown to confer resistance to adefovir (PMEA); MRP4 is associated with resistance to PMEG, cPr-PMEDAP and PMEDAP; and overexpression of MRP5 confers resistance to PMEDAP [39–43]. Today, none of these transporters has been studied in the context of CDV^R^, and we cannot exclude that upregulation of one of the MRPs might, in part, be responsible for CDV^R^ in SiHa_CDV_, HeLa_CDV_ and HaCaT_CDV_ cells. For instance, MRP2 is upregulated in SiHa_CDV_, and CDV was shown to be both a substrate and an inhibitor of this transporter in primary tubular renal cells [44]. BCRP (*ABCG2*) was found upregulated in SiHa_CDV_ while P-gp might be associated with HPMPA^R^ in HeLa_CDV_, since zosuquidar, a known inhibitor of P-gp, decreased CC_50_ value for HPMPA (Figure S8), even if no upregulation of P-gp was seen in HeLa_CDV_. These results suggest that altered levels of BCRP, MRP2 and P-gp transporters might contribute to CDV^R^ in SiHa_CDV_ and/or HeLa_CDV_.

On the other hand, CDV uptake has been described to be mediated by *SLC22A6*-encoded OAT1 and higher protein levels of OAT1 were found in SiHa_parental_ and HeLa_parental_ which may reduce CDV uptake. However, since fluid-phase endocytosis has been described as the major mechanism of CDV uptake, and the expression of SLC22A6 genes is predominantly observed in renal cells, the impact of altered OAT1 expression on intracellular CDV pools might be very limited [45].

The impaired activity of the UMP/CMPK1 enzyme in the SiHa_CDV_ and HeLa_CDV_ cells could explain, at least in part, their reduced growth rate. Since the R134M change destabilizes the structure of UMP/CMPK1 leading to a reduced amount of protein, the decrease in intracellular UTP and CTP pools in resistant cells may be responsible, at least in part, for their slower growth rate. Similarly, doubling times of HeLa_parental_ and HeLa_CDV_ cells were significant different suggesting an important role for UMP/CMPK1 in cellular growth. In HaCaT_CDV_, no significant decrease in UMP/CMPK1 protein level and no significant difference in growth rate were noted, even if CDV^R^ HaCaT cells appear to grow slightly faster than the parental counterpart. This might be the result of an increased GTP level in HaCaT_CDV_ compared to HaCaT_parental_ cells.

CDV^R^ seems to be acquired through alterations of CDV metabolism (i.e. impairment of UMP/CMPK1 activity) as well as MDR events (i.e. changes in expression of BCRP, MRP2 and OAT1) in SiHa and HeLa cells, indicating a multifactorial biochemical basis (Figure S8). On the other hand, in HaCaT cells, these factors were not found to be dramatically changed, suggesting that CDV^R^ is mediated through an alternate mechanism that might involve genes linked to DNA damage response. In these cells, the levels of intracellular CDV metabolites were unaltered, further excluding the enzymes involved in CDV activation as responsible for the resistance phenotype.

One may wonder whether the presence of a partial HPV genome and the constitutive expression of E6 and E7 oncoproteins might influence drug-resistance acquisition, since both SiHa_CDV_ and HeLa_CDV_, in contrast to HaCaT_CDV_ cells, underwent impairment of CDV activation and incorporation as well as alterations in the expression of drug transporters. Mutations in UMP/CMPK1 responsible for CDV-resistance have been selected and characterized in HPV(+) tumor cells and, to our knowledge, it is the first time that this enzyme has been involved in resistance against an antiviral or an anticancer drug, *in vitro* or *in vivo*. Up to now, no CDV^R^ has been observed in patients receiving CDV for HPV-associated diseases. Since UMP/CMPK1 is a key enzyme in the nucleoside salvage pathway, its activity cannot be substituted by another enzyme and therefore, the mutation-rate under drug-pressure might be very low. Hence, the high genetic barrier to CDV^R^ may be considered as an asset for the use of this drug in cancer therapy. On the other hand, a low number of patients suffering from HPV-associated diseases (genital warts or laryngeal papillomatosis) shows an uncomplete response to CDV therapy. Genotyping of *CMPK1* gene should be considered when CDV fails to clear HPV(+) cells in order to identify natural polymorphisms able to confer resistance to CDV.

The search for new drugs with anti-papillomavirus activity is urgently needed since, up to now, no chemotherapeutic agent has been brought to the market for the specific management of HPV-induced lesions. Although CDV has proven efficacious in the therapy of HPV-associated diseases, it has some limitations, the nephrotoxicity being the most encountered side-effect. Recent publications revealed some promising compounds such as the HIV-protease inhibitors lopinavir and nelfinavir, but further characterizations of their anti-HPV activity are needed [46]. Also, inhibition of cellular pathways frequently altered in HPV-induced neoplasia, such as phosphatidylinositol-3-kinase signaling represent a suitable target for therapeutic intervention [47].

In conclusion, acquisition of resistance to CDV in tumor cell appears to be a multifactorial process and different mechanisms are involved in distinct cell lines. Importantly, we showed that alterations in UMP/CMPK1 may contribute to CDV-resistance in some cell lines.

## MATERIALS AND METHODS

### Chemicals

The list of the ANPs and chemotherapeutics used in this study is provided in Tables S1 and Figure S9. The source of other chemical reagents was as follows: Uridine (Urd) and adenosine-5′-triphosphate (ATP) Calbiochem; cytidine (Cyd), Valeant Pharmaceuticals; cytidine-5′-monophosphate (CMP), uridine-5′-monophosphate (UMP), 2′-deoxycytidine-5′-monophosphate (dCMP) and 2′-deoxyuridine-5′-monophosphate (dUMP), Sigma-Aldrich; arabinocytidine-5′-monophosphate (araCMP), [5-^3^H]-radiolabeled CDV and [5-^3^H]-uridine, Moravek Biochemicals; and [5-^3^H]-radiolabeled cytidine, MP Biochemicals.

### Selection of CDV^R^ cells and growth rate

SiHa [HPV16(+)] and HeLa [HPV18(+)] cells were obtained from ATCC (#HTB-35 and #CCL-2, respectively) (Manassas, USA). HaCaT [HPV(−)] cells were kindly provided by F. De Marco (Regina Elena Institute for Cancer Research, Rome, Italy). Cell cultures were maintained in Dulbecco's modified Eagle's medium supplemented with 10% fetal calf serum. Selection of HeLa_CDV_ and HaCaT_CDV_ cells was performed as previously described for SiHa_CDV_ cells [48]. The growth rate of CDV^R^ cells compared to parental cells was evaluated as previously reported [48]. Doubling time (DT) was calculated with the formula: DT = (t_2_–t_1_)/(log_2_N_2_-log_2_N_1_), where t_1_ and t_2_ are the times at which the cells were counted, and N_1_ and N_2_ are the cells numbers at times t_1_ and t_2_.

### Microarray experiments

Parental and CDV^R^ cells were allowed to grow for 72 h in medium without CDV. Total RNA of 1 × 10^6^ cells was isolated with TRIzol reagent (Invitrogen) and further purified by RNeasy Mini Kit (Qiagen). RNA quality and quantity were assessed with a Bioanalyzer system (Agilent).

Microarray data were generated as previously reported [12] and is deposited in the Gene Expression Omnibus (GEO, http://www.ncbi.nlm.nih.gov/projects/geo) according to MIAME standards under accession number GSE39293: http://www.ncbi.nlm.nih.gov/geo/query/acc.cgi?acc=GSE39293.

Bioinformatics analysis of differentially expressed (DE) genes was carried out with Ingenuity Pathways Analysis (IPA, Ingenuity^®^ Systems) version 9 as previously reported [12].

### Antiproliferative activity

Inhibition of cell growth was determined following 7 days of incubation with the assessed drug as previously described [49]. The antiproliferative effects were expressed as CC_50_ (50% cystostatic concentration), which is the concentration required to reduce cell growth by 50% relative to the number of cells in untreated control cell culture). Fold-resistance (FR) was calculated as the ratio of CC_50_ for CDV^R^ cells to CC_50_ for parental cells.

### Genotyping of UMP/CMPK1, UMP/CMPK2, HPV E6 and E7

Total mRNA from parental and CDV^R^ cells was isolated with the Quickprep mRNA purification kit (Amersham Biosciences) and converted to cDNA with the first-strand cDNA synthesis kit (GE Healthcare). The entire cDNA from each selected gene was amplified by PCR using specific primers. The PCR products were purified using PCR product purification kit (Roche) and directly sequenced using a cycle-sequencing kit (Dyenamic dye terminator kit; Amersham Biosciences), specific primers targeting both strands of the specific gene, and a capillary DNA sequencing system (MegaBACE 500; Amersham Biosciences). The data were assembled and compared to the DNA sequences obtained from reference sequences using Vector NTI software (Invitrogen). The primers used for the genotyping of UMP/CMPK1 and 2, and HPV oncogenes E6 and E7 are listed in Table S2.

### CDV metabolism

[5-^3^H]-radiolabeled CDV (Moravek) was used to assess the metabolism and drug incorporation into cellular nucleic acid material in parental and CDV^R^ cells. The assay was performed with 10 μM of CDV for 24 h, a protocol adapted to the one described previously [12]. All experiments were performed in duplicate.

### Measurement of intracellular nucleotide levels

To quantify the levels of CMP, CDP, CTP and UTP, semi-confluent parental and CDV^R^ cells were grown in presence or absence of 100 μM of uridine (Urd) and cytidine (Cyd) for 72 h. Cells were trypsinized, collected by centrifugation, and subjected to cell lysis. To determine the nucleotide pools, the extracts were submitted to HPLC analysis on an anion exchange column [PartiSphere SAX column (4.3 mm × 125 mm), Whatman] followed by UV spectroscopy. In parallel, 5 μCi per flask of radioactive [5-^3^H] labeled Urd or Cyd, supplemented in the growth medium of the parental and CDV^R^ cells were used to investigate UTP and CTP biosynthesis at 6 h and 24 h after addition of the nucleosides.

### Site-directed mutagenesis of UMP/CMPK1

The P64T, R134M and P64T/R134M changes were introduced in the pHL60-5 plasmid containing the UMP/CMPK1 gene with a three step protocol using the Quickchange Lightning Multi site-directed Mutagenesis kit (Agilent technologies^®^). Primer(s) (Table S2) containing the base change(s) were used to perform a PCR reaction in order to produce mutant strands prior to enzymatic digestion of the template with *Dpn* I. To amplify the mutated plasmids, XL10 gold ultracompetent cells were transformed. Sequencing of the full UMP/CMPK1 gene from the recombinant plasmid was performed on both strands to exclude any additional mutation.

### Expression, purification and enzymatic activity measurement of recombinant UMP/CMPKs

The wild-type and mutant UMP/CMPK1s were produced in *Escherichia coli* [strain BL21 Rosetta^®^ (DE3) pLysS (Novagen)] as previously reported [14]. For protein purification, cells were broken by sonication and centrifuged for 30 min at 15,000 g and 4°C. The supernatant was added onto a column containing 2 ml Ni-Nitrilotriacetic acid resin (Ni-NTA) (Qiagen, Benelux). Protein elution was performed using increasing concentrations of imidazol and fractions were collected and pooled for dialysis. Protein purity was analyzed by polyacrylamide gel electrophoresis (SDS-PAGE) in denaturing conditions. The catalytic activity of the recombinant wild-type and mutant forms of human UMP/CMPK1 was determined using a previously described method [16] based on ADP formation [50].

### Circular dichroism

All circular dichroism (CD) spectra measurements were acquired using a JOBIN-YVON CD6 spectropolarimeter as previously described [51]. CD measurements are reported as Δε (M^−1^.cm^−1^). The relative helix content was deduced according to Zhong and Johnson as the percent of helix = [Δε_222nm_ × −10], where Δε_222nm_ is the dichroic increment at 222 nm per residue [52]. Thermal denaturation curves of UMP/CMPK1s were obtained by monitoring Δε at 222 nm as a function of temperature from 0°C to 70°C with 10°C temperature steps. Experiments were performed in triplicate.

### Structural model for the amino acid changes in human UMP-CMPK1

The role of the amino acid changes identified in the human UMP-CMPK1 in SiHa_CDV_ cells was investigated by building a model of the enzyme complexed to ADP and CMP based on the published three-dimensional structure of the *Dictyostelium discoideum* UMP-CMPK (pdb code: 2UKD). Pymol Delano software was used to introduce the identified mutations and to visualize the generated models. A comparison of the open conformation of the human enzyme free of substrate (pdb code: 1TEV) and the closed conformation obtained by modeling was performed to predict the impact of the P64T mutation. All figures were generated using PyMol software.

### Western blot analysis

UMP/CMPK1 and 2 were detected, respectively, using a mouse monoclonal antibody (ab77457) and a rabbit polyclonal antibody (ab103658) (Abcam, Cambridge, UK). Detection of the transporters P-glycoprotein (ab3083), MRP2 (ab3373), BCRP (ab130244) and OAT1 (ab118346) was performed on total cell membrane extracts with Plasma membrane protein extraction kit (ab65400). The house-keeping gene actin was detected using a mouse monoclonal antibody (ab3280). Species-specific HRP-conjugated secondary antibodies (Dako, Denmark) were used, in combination with Supersignal West Pico or Femto Chemiluminescent substrate (Thermo Scientific, Rockford, IL, USA)

### Statistical analysis

Plotting and statistical analysis were performed using GraphPad software. The unpaired *t*-test was used to analyze several parameters between parental and CDV^R^ cells (i.e. intracellular CDV metabolites, drug transporter protein levels, UMP/CMPK protein levels and drug-sensitivity). Comparison of CTP and UTP biosynthesis in parental and CDV^R^ cells was carried out using the two-way analysis of variance test (two-way ANOVA). No correction for multiple testing was used due to the large number of statistical analyses performed. Data are presented as mean ± standard deviation (SD) and *p* < 0.05 was considered significant.

## SUPPLEMENTARY MATERIALS TABLES AND FIGURES


