# Supplementary material for: Resistance to the nucleotide analogue cidofovir in HPV(+) cells: a multifactorial process involving UMP/CMP kinase 1
Source: Oncotarget. 2016 Jan 25;7(9):10386–401. doi: 10.18632/oncotarget.7006 (PMC4891127; doi:10.18632/oncotarget.7006)
Supplement: Supplementary file 1 [file oncotarget-07-10386-s001.pdf]

Resistance to the nucleotide analogue cidofovir in HPV(+) cells: a multifactorial process involving UMP/CMP kinase 1

Supplementary Materials

Supplementary Table S1: Chemical compounds tested in this study (the structure of these drugs is in depicted Figure S9)

Group	Designation(s) of compound	Description
First class ANP	HPMPC, cidofovir, CDV, Vistide® **(1)**	(S)-1-[3-hydroxy-2-(phosphonomethoxy)propyl]cytosine
	cHPMPC, cyclic cidofovir, cCDV **(2)**	Cyclic (S)-HPMPC
	HPMPA **(3)**	(S)-9-[3-hydroxy-2-(phosphonomethoxy)propyl]adenine
	cHPMPA **(4)**	Cyclic (S)-HPMPA
	3-deaza-HPMPA **(5)**	(S)-9-[3-hydroxy-2-(phosphonomethoxy)propyl]-3-deazaadenine
	HPMPDAP (6)	(S)-9-[3-hydroxy-2-(phosphonomethoxy)propyl]-2,6-diaminopurine
	PMEA, adefovir, Hepsera® **(7)**	9-[2-(phosphonomethoxy)ethyl]adenine
	PMEDAP **(8)**	9-[2-(phosphonomethoxy)ethyl]-2,6-diaminopurine
	PMEG **(9)**	9-[2-(phosphonomethoxy)ethyl]guanine
	cPr-PMEDAP **(10)**	9-[2-(phosphonomethoxy)ethyl]-N^6-cyclopropyl-2,6-diaminopurine
Second class ANP	HPMPO-DAPY **(11)**	(R)-{2,4-diamino-3-hydroxy-6-[2-(phosphonomethoxy)propyl]} pyrimidine
	PMEO-DAPY **(12)**	2,4-diamino-6-[2-(phosphonomethoxy)ethoxy]pyrimidine
Third class ANP	HPMP-5-azaC **(13)**	(S)-1-[3-hydroxy-2-(phosphonomethoxy)propyl]-5-azacytosine
	cHPMP-5-azaC **(14)**	Cyclic HPMP-5-azaC
Microtubule inhibitors	Vincristine **(a)**	22-oxovincaleucoblastine
	Docetaxel, taxotere **(b)**	1,7β,10β-trihydroxy-9-oxo-5β,20-epoxytax-11-ene-2α,4,13α-triyl 4-acetate 2-benzoate 13-{(2R,3S)-3-[(tert-butoxycarbonyl)amino]-2-hydroxy-3-phenylpropanoate}
Anti-metabolites	Methotrexate **(c)**	(2S)-2-[(4-{[(2,4-diaminopteridin-6-yl)methyl](methyl)amino} benzoyl)amino]pentanedioic acid
	Fludarabine **(d)**	[(2R,3R,4S,5R)-5-(6-amino-2-fluoro-purin-9-yl)- 3,4-dihydroxy-oxolan-2-yl]methoxyphosphonic acid
	5-Fluorouracil, 5-FU **(e)**	5-fluoro-1H,3H-pyrimidine-2,4-dione
	Cytarabine, araC **(f)**	4-amino-1-[(2R,3S,4R,5R)-3,4-dihydroxy-5- (hydroxymethyl) oxolan-2-yl] pyrimidin-2-one
	Gemcitabine **(g)**	4-amino-1-(2-deoxy-2,2-difluoro-β-D-erythro-pentofuranosyl) pyrimidin-2(1H)-on
	Hydroxyurea **(h)**	Hydroxyurea

**Topoisomerase inhibitors**	Camptothecin **(i)**	(S)-4-ethyl-4-hydroxy-1H-pyrano[3′,4′:6,7]indolizino[1,2-b] quinoline-3,14-(4H,12H)-dione
	SN-38 **(j)**	7-Ethyl-10-hydroxy-camptothecin
	Topotecan **(k)**	(S)-10-[(dimethylamino)methyl]-4-ethyl-4,9-dihydroxy-1H-pyrano [3′,4′:6,7] indolizino [1, 2-b]quinoline-3,14(4H,12H)-dione monohydrochloride
	Etoposide **(l)**	4′-Demethyl-epipodophyllotoxin 9-[4,6-O-(R)-ethylidene-beta-D-glucopyranoside], 4′ -(dihydrogen phosphate)
**DNA damage inducers**	Daunorubicin **(m)**	(8S,10S)-8-acetyl-10-[(2S,4S,5S,6S)-4-amino-5-hydroxy-6-methyl-oxan-2-yl]oxy-6,8,11-trihydroxy-1-methoxy-9,10-dihydro-7H-tetracene-5,12-dione
	Cis-platin **(n)**	(SP-4-2)-diamminedichloroplatinum(II)
	Bleomycin **(o)**	(3-{[(2′-{(5S,8S,9S,10R,13S)-15-{6-amino-2- [(1S)-3-amino-1-{[(2S)-2,3-diamino-3-oxopropyl]amino}-3-oxopropyl] -5-methylpyrimidin-4-yl}-13-[{[(2R,3S,4S,5S,6S)-3-{[(2R,3S,4S,5R,6R)-4-(carbamoyloxy)-3,5-dihydroxy-6-(hydroxymethyl)tetrahydro-2H-pyran-2-yl]oxy} -4,5-dihydroxy-6-(hydroxymethyl)tetrahydro-2H-pyran-2-yl]oxy} (1H-imidazol-5-yl)methyl]-9-hydroxy-5-[(1R)-1-hydroxyethyl]-8,10-dimethyl-4,7,12,15-tetraoxo-3,6,11,14-tetraazapentadec-1-yl}-2,4′-bi-1,3-thiazol-4-yl)carbonyl]amino}propyl)(dimethyl)sulfonium
**EGFR inhibitors**	Canertinib, CI-1033 **(p)**	N-{4-[(3-Chloro-4-fluorophenyl)amino]-7-[3-(morpholin-4-yl) propoxy]quinazolin-6-yl}prop-2-enamide
**P-glycoprotein inhibitors**	Zosuquidar **(A)**	(2R)-1-{4-[(1aR,10bS)-1,1-difluoro-1,1a,6,10b-tetrahydrodibenzo [a,e]cyclopropa[c][7]annulen-6-yl}-3-(quinolin-5-yloxy)propan-2-ol
	Tariquidar **(B)**	N-[2-[[4-[2-(6,7-Dimethoxy-3,4-dihydro-1H-isoquinolin-2-yl)ethyl] phenyl]carbamoyl]-4,5-dimethoxyphenyl]quinoline-3-carboxamide
**MRP inhibitors**	Reversan **(C)**	N-[3-(4-Morpholinyl)propyl]-5,7-diphenyl-pyrazolo[1,5-a] pyrimidine-3-carboxamide
	MK-571 **(D)**	5-(3-(2-(7-Chloroquinolin-2-yl)ethenyl)phenyl)-8-dimethylcarbamyl-4,6-dithiaoctanoic acid
**COX-1 & -2 inhibitor**	Indomethacin **(E)**	2-{1-[(4-Chlorophenyl)carbonyl]-5-methoxy-2-methyl-1*H*-indol-3-yl}acetic acid
**Ca^{2+} channel blocker**	Verapamil **(F)**	(RS)-2-(3,4-dimethoxyphenyl)-5-{[2-(3,4-dimethoxyphenyl)ethyl]-(methyl)amino}-2-prop-2-ylpentanenitrile

Supplementary Table S2: (A) Oligonucleotides used for the sequencing of the UMP/CMPK1, UMP/CMPK2, HPV16 E6 and E7, HPV18 E6 and E7 genes

Targeted gene	Oligonucleotides name	Sequence (5′-to-3′)
**UMP/CMPK1**	CMPK1_F29	TCCACGTCCTGGGCCTTAGCTT
	CMPK1_F310	TTAAAGAGGGAAATGGATCAGACAA
	CMPK1_R431	GCCTTCCCATCCATGGTCTTG
	CMPK1_R681	TTCCTTGTCAAAAATCTGCACAACT
**UMP/CMPK2**	CMPK2_97F	TTCGTCCTGGAGCTTCCCGA
	CMPK2_116F*	ACTGCACCCTGGCTCACTT
	CMPK2_335F	TGCTCAGGCTGCTCTGCTACT
	CMPK2_336F*	GCTCAGGCTGCTCTGCTACT
	CMPK2_607F	GTGGTGCCAGACTTGCCCAGTT
	CMPK2_752F*	AGTTCCAGGTTGTTGCCATC
	CMPK2_986F	TAGACAGGTACTGGCACAGCACG
	CMPK2_432R	TTGCCGGGTGTCAGGGTCAT
	CMPK2_699R*	GGCTTCAGGAATAAAGGAGGT
	CMPK2_822R	ATCTGCCACTGACTGGGTCACC
	CMPK2_844R*	ATGCAAGAGGGTGGTGACTT
	CMPK2_1156R	GCCTCTGCAACCTCTCCTCA
	CMPK2_1306R	GCAGGACCTTTTCTCTGGAGG
	CMPK2_1382R*	CGTCTGCAGGACCTTTTCTC
**HPV16 E6–E7**	HPV16_F1	TGGGTTACACATTTACAAGC
	HPV16_F2	GAGCGACCCAGAAAGTTACCAC
	HPV16_F3	GAACAGCAATACAACAAACCGT
	HPV16_F4	CAGAGGAGGAGGATGAAATAG
	HPV16_R1	AAATCCCGAAAAGCAAAGTC
	HPV16_R2	TTGTCCAGATGTCTTTGCTT
	HPV16_R3	CACAACCGAAGCGTAGAGTC
	HPV16_R4	CCACTACAGCCTCTACATAA
**HPV18 E6–E7**	HPV18_F1	GTAACCGAAAACGGTCGGGA
	HPV18_F2	TAATAAGGTGCCTGCGGTGC
	HPV18_R1	TTGGAGTCGTTCCTGTCGTG
	HPV18_R2	GTTGCTTACTGCTGGGATGC

(*) Alternative primers used for the sequencing of CMPK2 gene from parental and CDV-resistant HeLa and HaCaT cells.

Supplementary Table S2: (B) Oligonucleotides used for the site-directed mutagenesis on UMP/CMPK1 gene

Oligonucleotides name	Sequence (5′-to-3′)
UMP/CMPK1_P64T_Fwd	GAAGGAAAGATTGTAACAGTTGAGATAACCATCAG
UMP/CMPK1_P64T_Fwd_DoubleMut	GGAAAGATTGTAACAGTTGAGATAACC
UMP/CMPK1_R134M_Fwd	CGATGTCTTGAGATGGGAAAGAGTAGTGG

An alternative primer of UMP/CMPK_P64T has been used for the generation of the double mutant P64T + R134M. The bases in bold and underlined are indicating the mutated position.

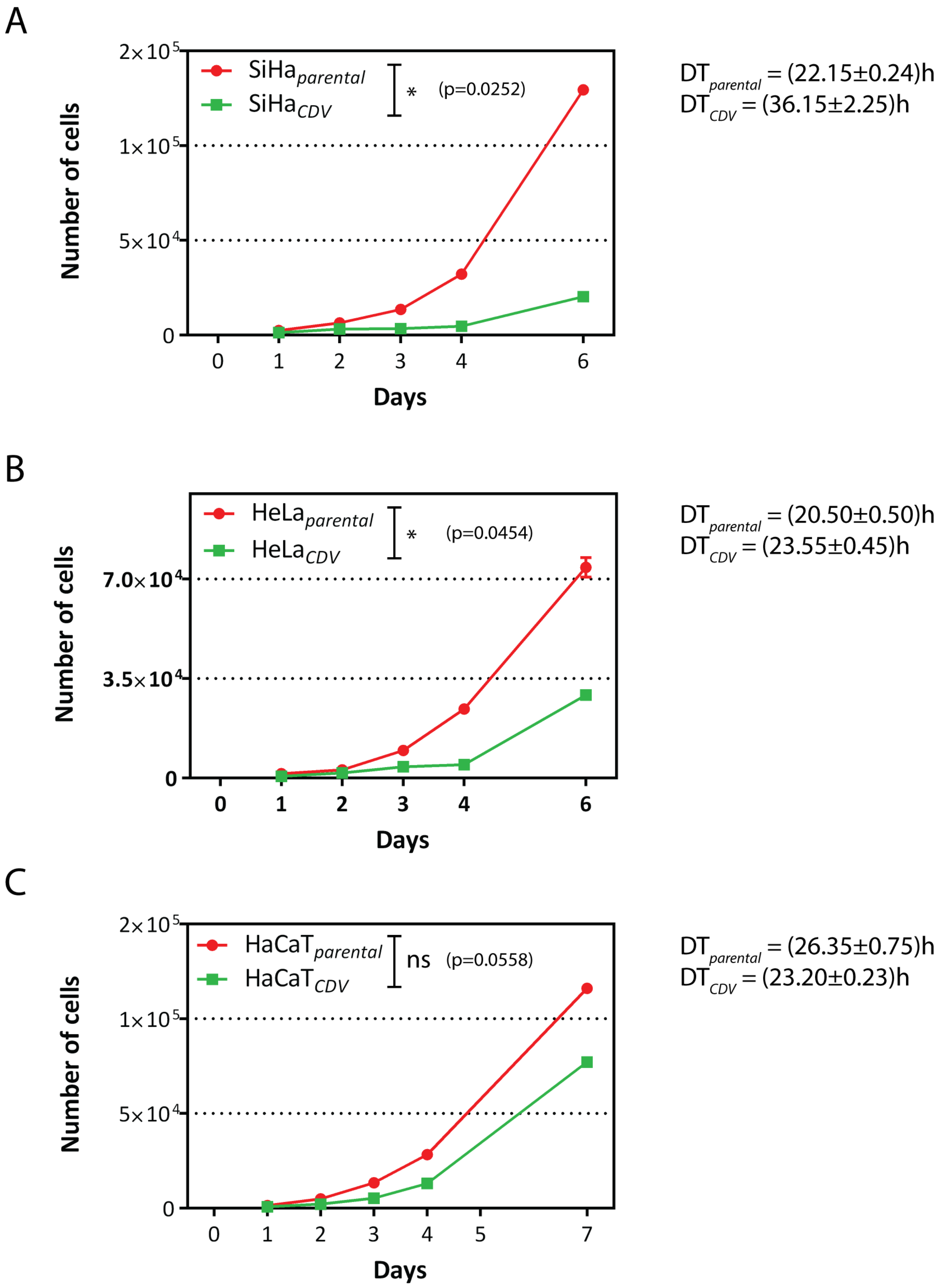

Supplementary Figure S1: Growth rate of CDV-resistant SiHa, HeLa and HaCaT cells compared to parental cells. Doubling time (DT) was calculated as described in the Materials and Methods section.

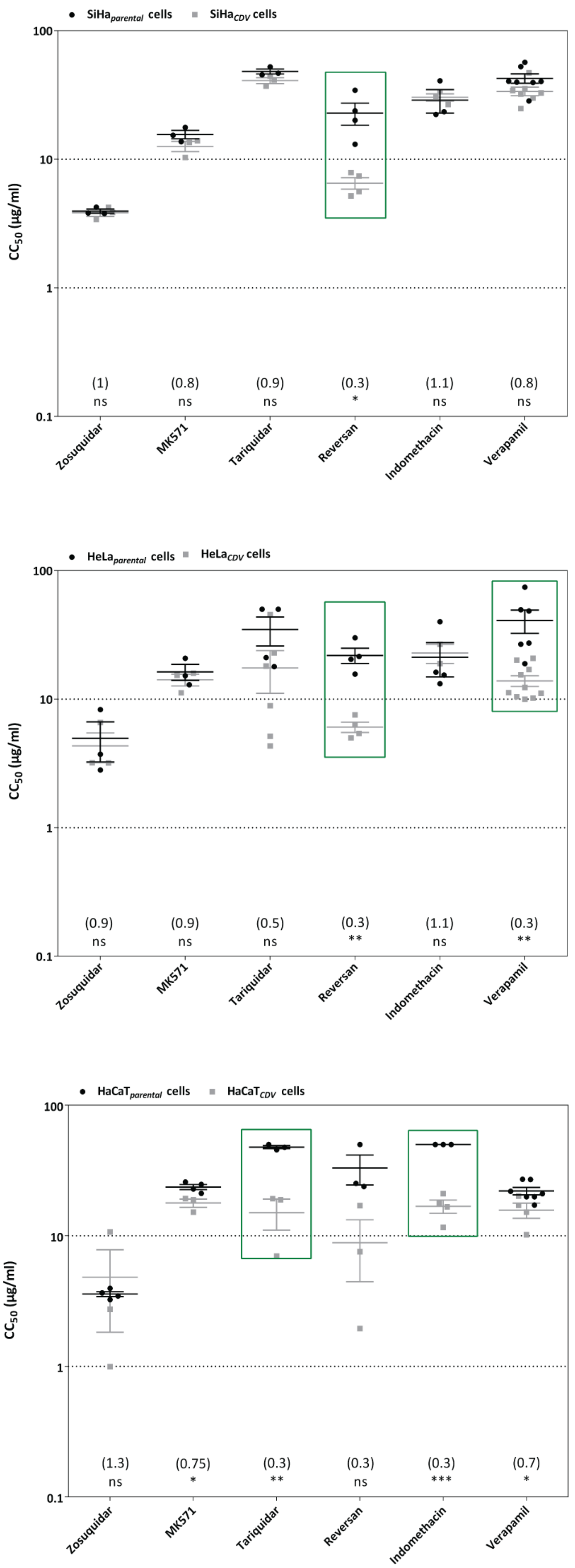

Supplementary Figure S2: antiproliferative activity of several MDR inhibitor on $SiHa_{parental}$, $SiHa_{CDV}$, $HeLa_{parental}$, $HeLa_{CDV}$, $HaCaT_{parental}$ and $HaCaT_{CDV}$.

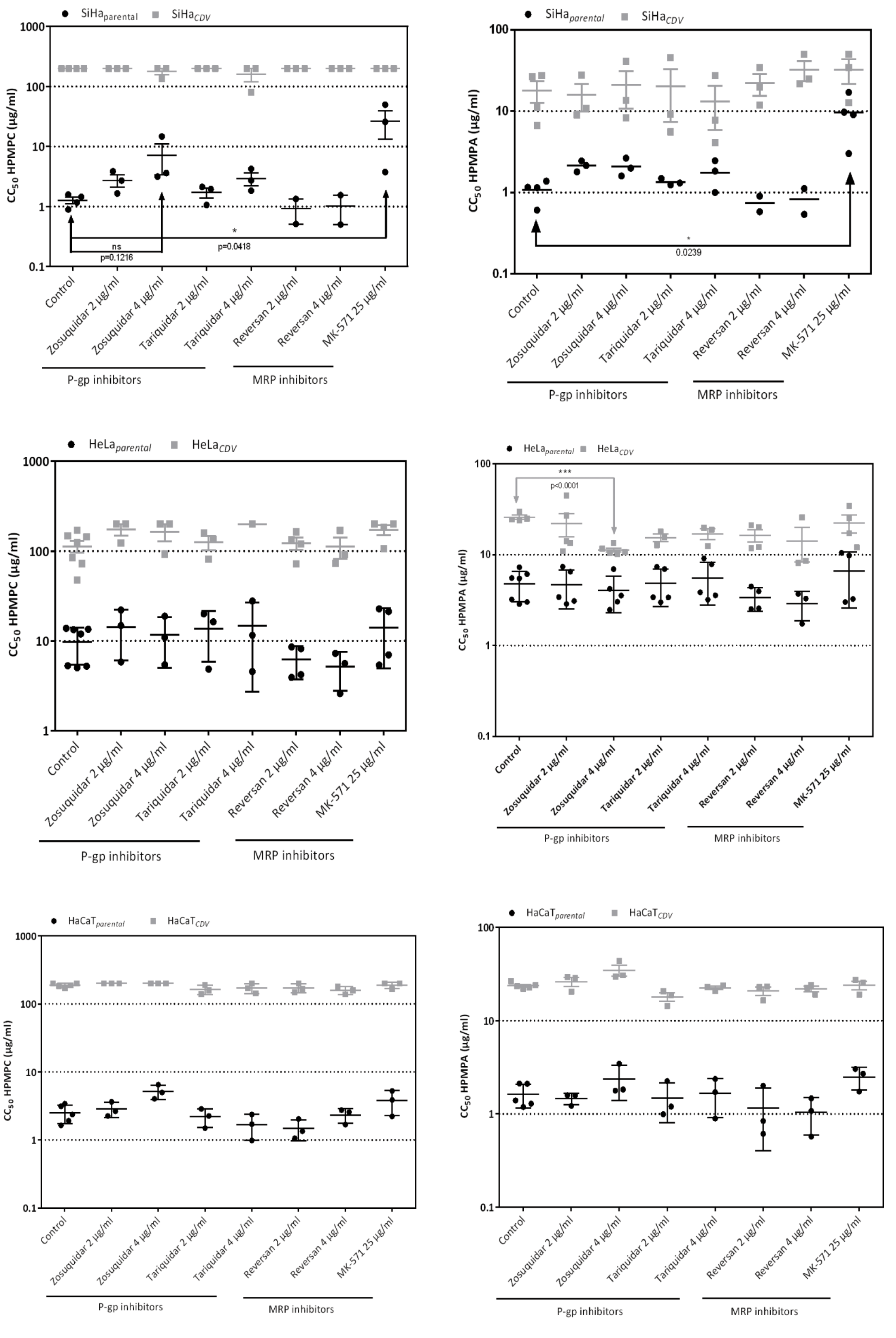

Supplementary Figure S3: antiproliferative activity of CDV and HPMPA on SiHa$_{parental,}$ SiHa$_{CDV,}$ HeLa$_{parental,}$ HeLa$_{CDV,}$ HaCaT$_{parental}$ and HaCaT$_{CDV}$ in the presence of several MDR Inhibitors.

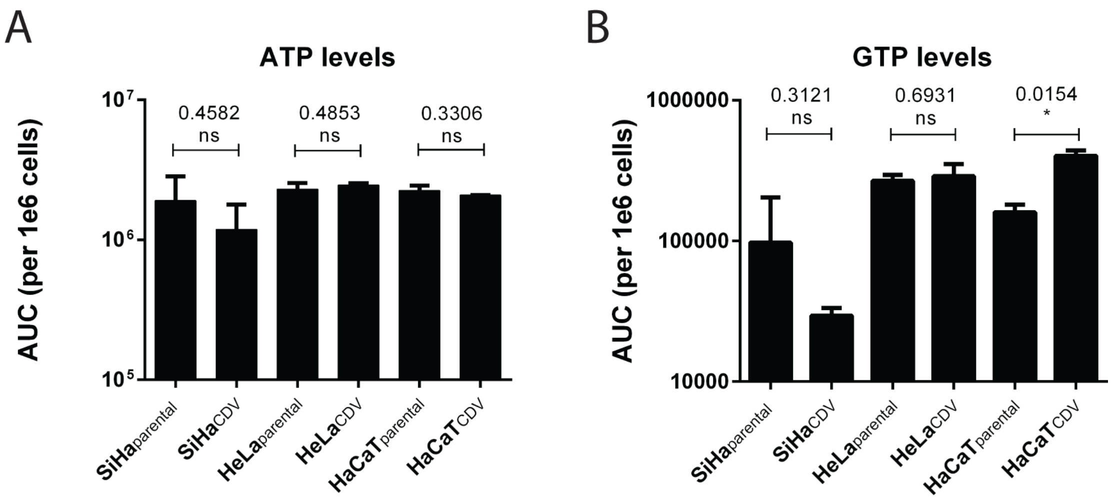

Supplementary Figure S4: ATP and GTP levels measured by means of HPLC in parental and CDV[R] tumor cell lines. Statistical significance was assessed using an unpaired *t*-test.

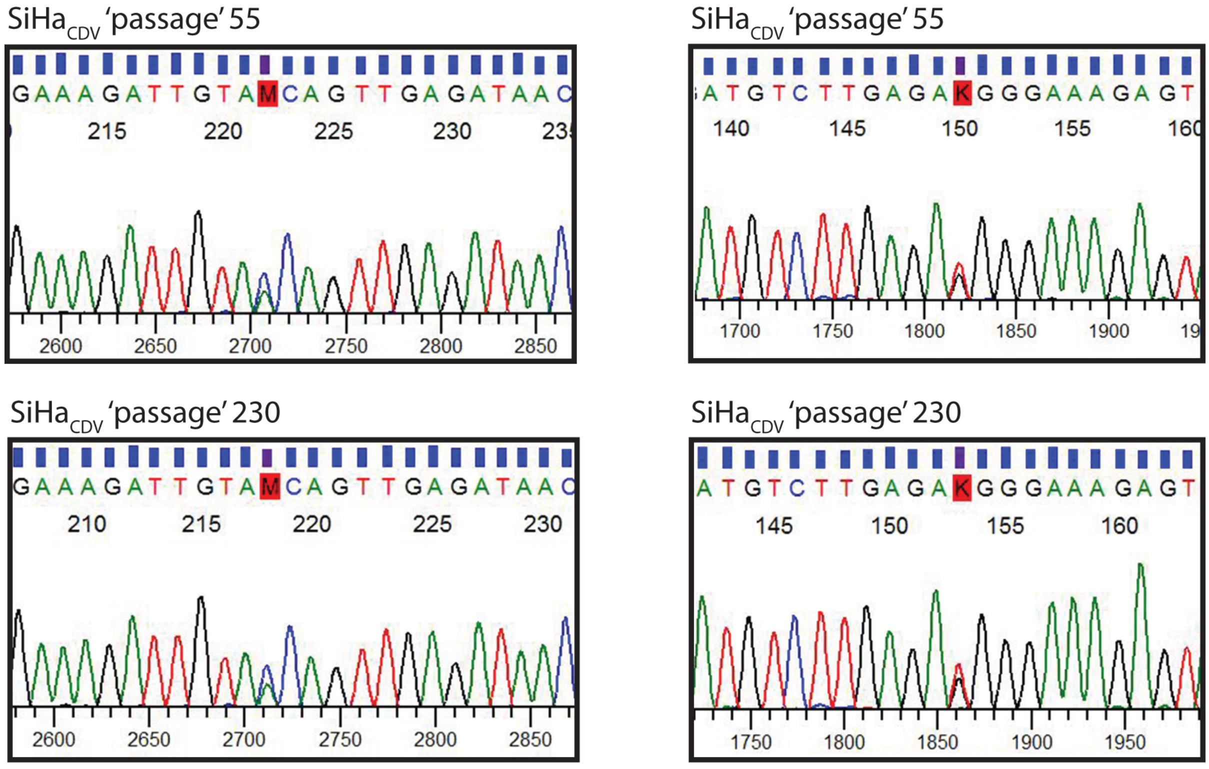

A--->A/C P64 ----> P64/T64

G--->G/T R134 ----> R134/M134

Supplementary Figure S5: electropherograms showing the identified mutations in *CMPK1* gene of SiHa$_{CDV}$ at passage #55 and #230.

A

```
                               P-loop                      NMP binding domain
WT  UMP/CMPK1     MKPLVVFVLGGPGAGKGTQCARIVEKYGYTHLSAGELLRDERKNPDSQYGELIEKYIKEGK   61
Mut UMP/CMPK1     MKPLVVFVLGGPGAGKGTQCARIVEKYGYTHLSAGELLRDERKNPDSQYGELIEKYIKEGK   61
                     64
WT  UMP/CMPK1     IVPVEITISLLKREMDQTMAANAQKNKFLIDGFPRNQDNLQGWNKTMDGKADVSFVLFFDC   122
Mut UMP/CMPK1     IVTVEITISLLKREMDQTMAANAQKNKFLIDGFPRNQDNLQGWNKTMDGKADVSFVLFFDC   122
                              134    LID domain
WT  UMP/CMPK1     NNEICIERCLERGKSSGRSDDNRESLEKRIQTYLQSTKPIIDLYEEMGKVKKIDASKSVDE   183
Mut UMP/CMPK1     NNEICIERCLEMGKSSGRSDDNRESLEKRIQTYLQSTKPIIDLYEEMGKVKKIDASKSVDE   183

WT  UMP/CMPK1     VFDEVVQIFDKEG------------------------------------------------   196
Mut UMP/CMPK1     VFDEVVQIFDKEG------------------------------------------------   196
```

B

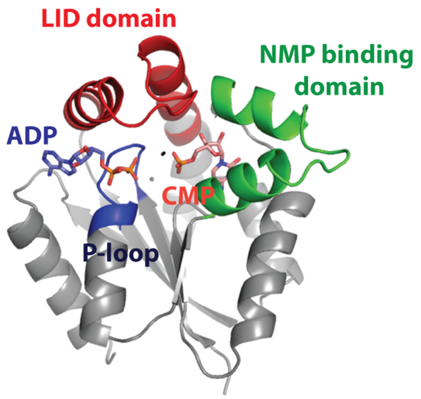

Supplementary Figure S6: (**A**) sequence alignment of the wild-type and the mutant UMP/CMPK1. The main domains of the UMP/CMPK1 are represented by colored boxes. Mutated positions 64 and 134 are shown as red bold letter. (**B**) Model of the human UMP/CMPK1 in closed conformation, complexed to CMP and ADP (based on the structure of the *D.discoideum* UMP/CMPK; pdb code: 2UKD). The same colors are used, as in panel A, to depict the domains of the proteins.

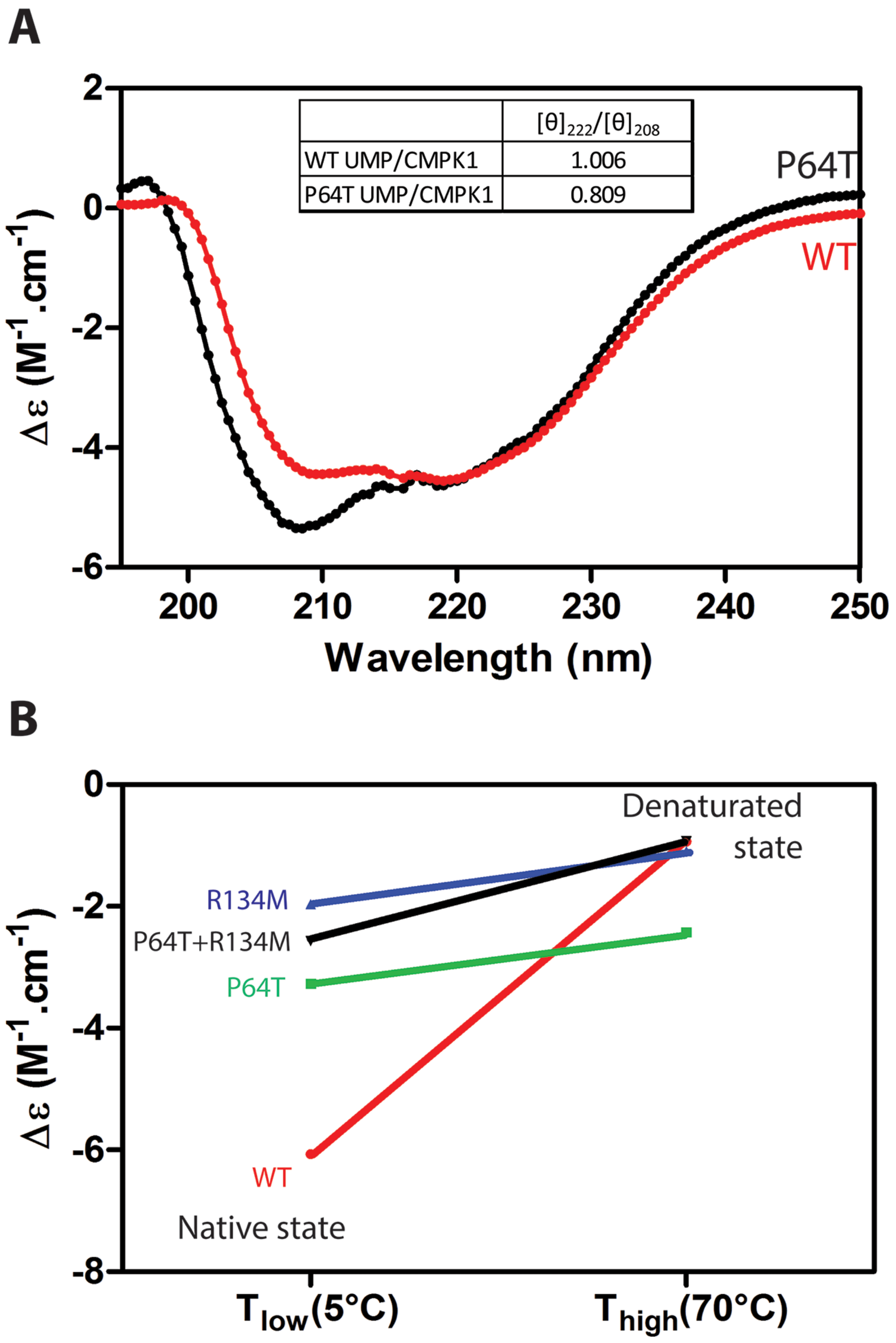

Supplementary Figure S7: (**A**) circular dichroism spectra of the wild-type and the P64T UMP/CMPK1 followed between 195 nm and 250 nm. The spectra were acquired at 25°C in Tris-HCl buffer. Molar ellipticity for both proteins was calculated as the ratio of $\theta_{222}/\theta_{208}$. (**B**) Native and denaturated states of the wild-type and the mutants UMP/CMPK1s at the extreme temperatures of 5°C and 70°C.

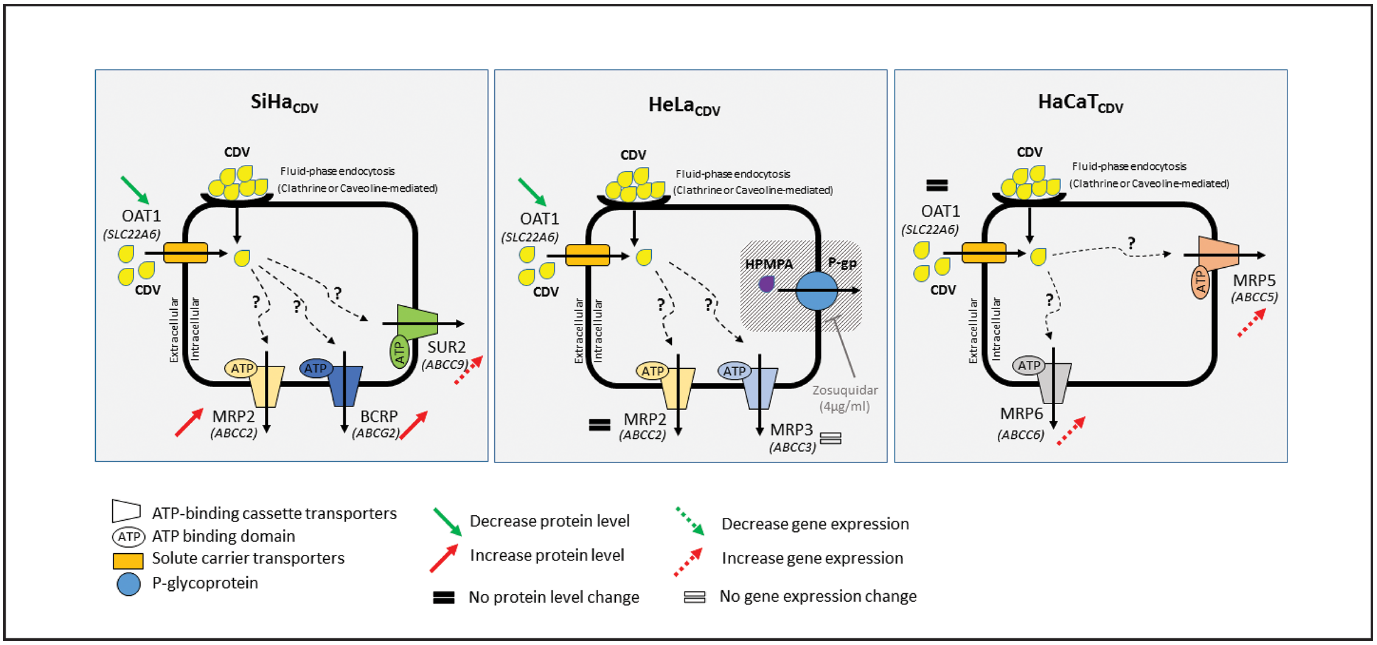

Supplementary Figure S8: Scheme summarizing the efflux transporters that might be linked with CDV^R in the three cell types. Uptake of CDV is shown as fluid-phase endocytosis or through OAT1 that have been described in the literature. Hypothesis have been made on the efflux through the different ABC transporters according to the data obtain by microarray experiments. In SiHa$_{CDV}$ cells, MRP2, BCRP and SUR2 are good candidates to validate CDV^R through drug efflux. In HeLa$_{CDV,}$ MRP2 and MRP3 are upregulated and might contribute to CDV^R. It is worth noting that P-gp is probably involved in HPMPA^R since co-treatment with zosuquidar is able to revers resistance to HPMPA. In HaCaT$_{CDV,}$ MRP5 and MRP6 might be involved in CDV^R.

Acyclic nucleoside phosphonates

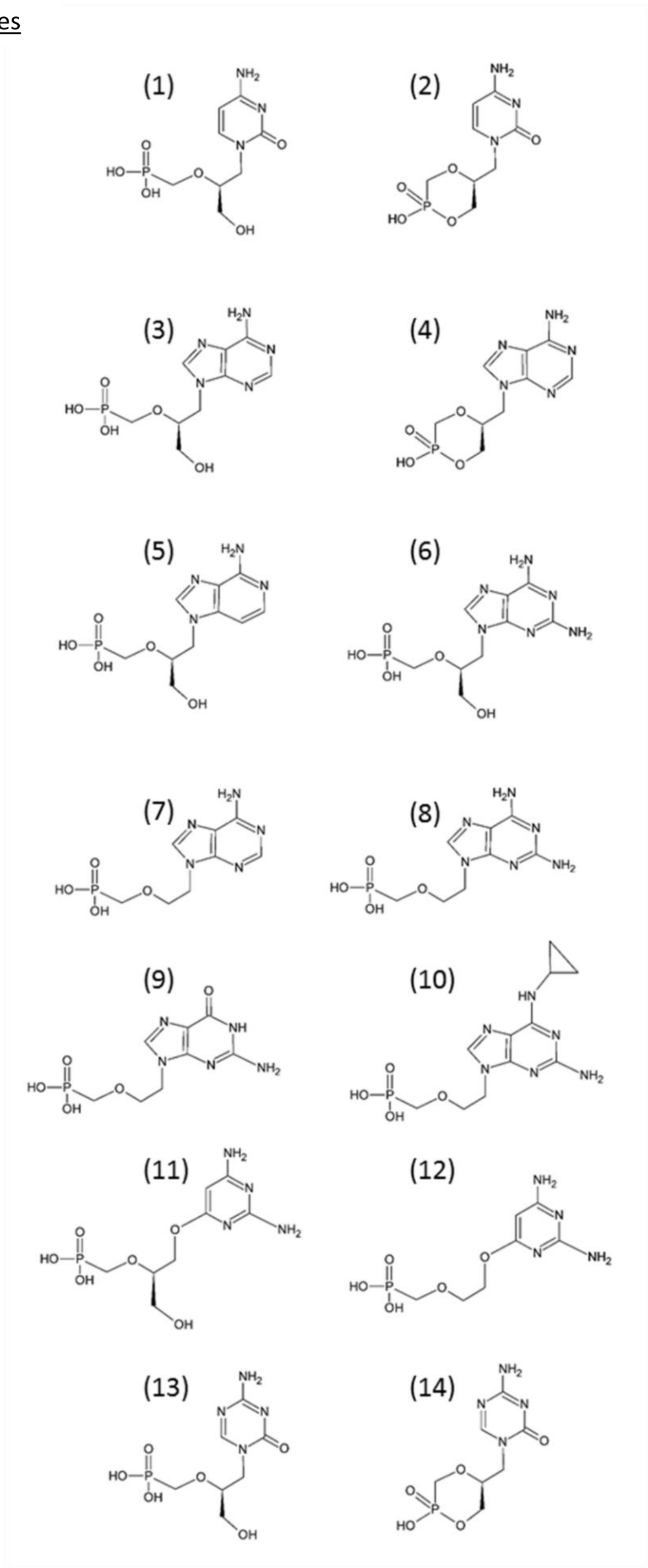

Anticancer chemotherapeutics :

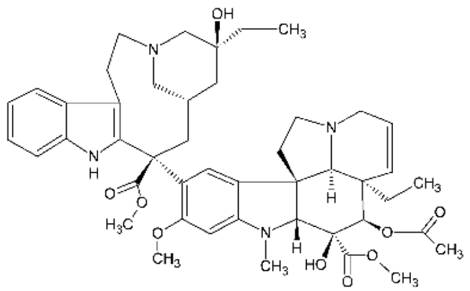

(a)

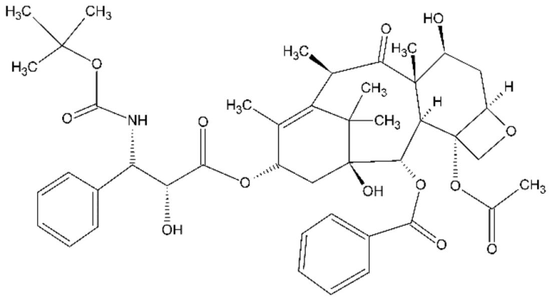

(b)

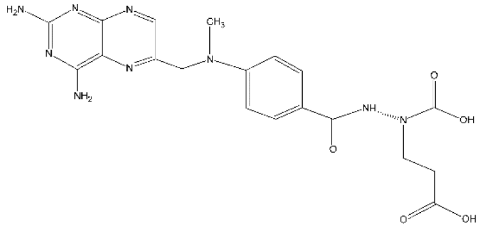

(c)

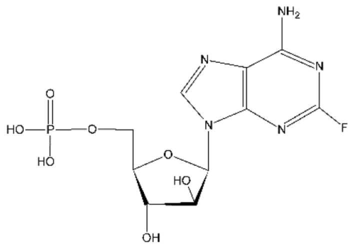

(d)

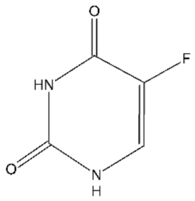

(e)

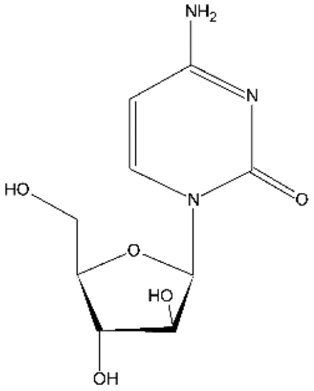

(f)

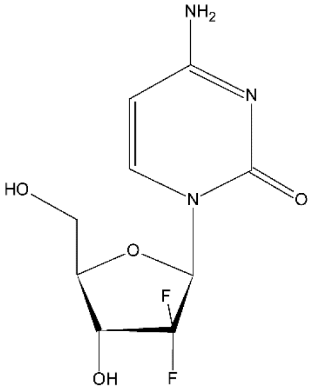

(g)

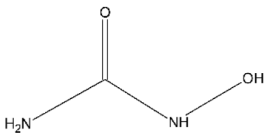

(h)

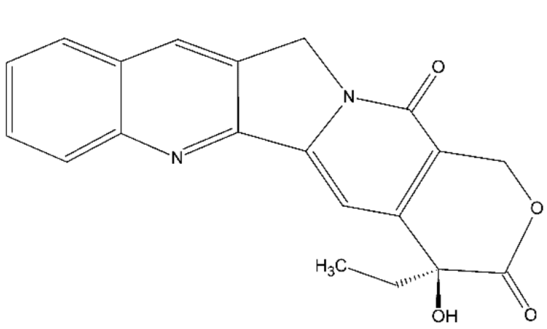

(i)

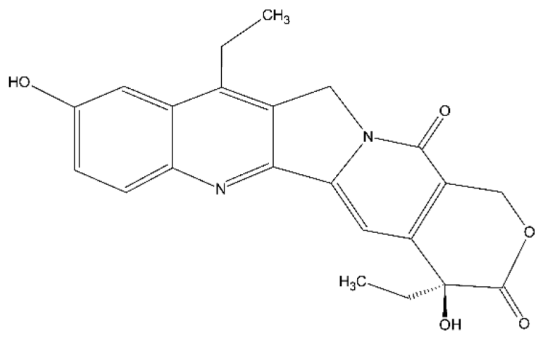

(j)

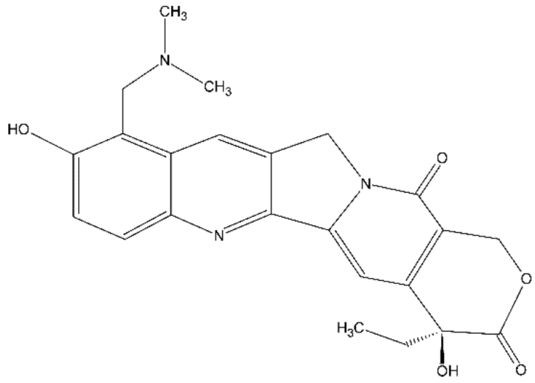

(k)

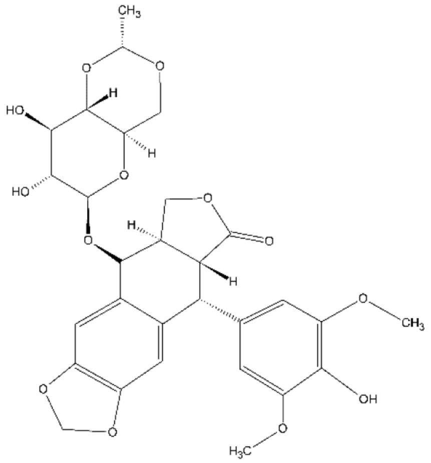

(l)

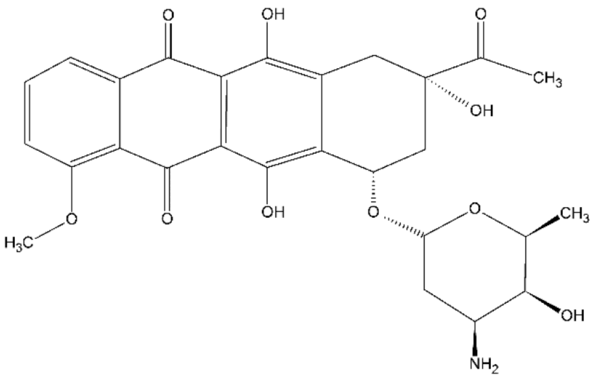

(m)

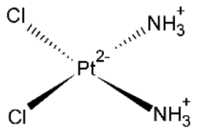

(n)

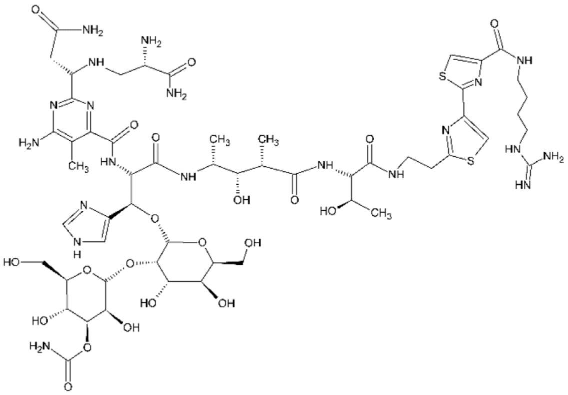

(o)

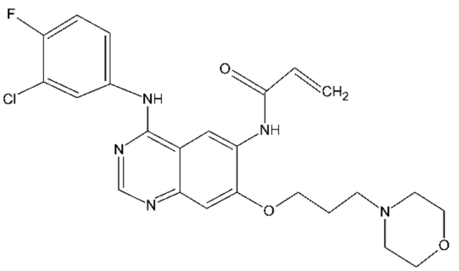

(p)

Transporters inhibitors

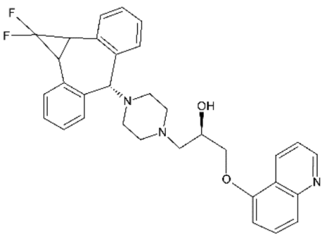

(A)

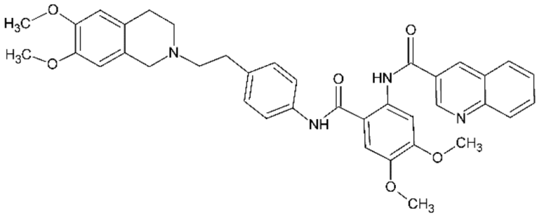

(B)

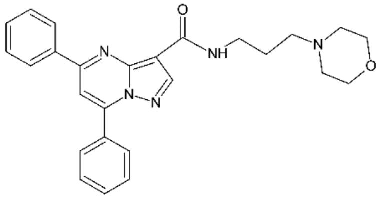

(C)

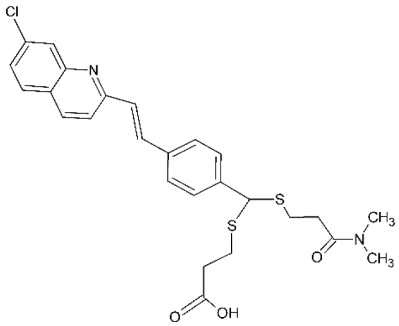

(D)

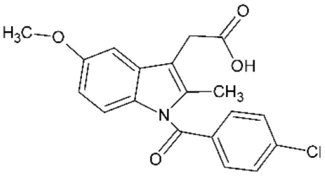

(E)

Supplementary Figure S9: Chemical structure of the compounds used in this study. Compounds (**1–14**) correspond to the nucleotide analogs. (**a–p**) groups the drugs used in cancer therapy and (**A–E**) are the transporter inhibitors assessed in this study.
